# Comparative Genomics of *Mycoplasma bovis* Strains Reveals That Decreased Virulence with Increasing Passages Might Correlate with Potential Virulence-Related Factors

**DOI:** 10.3389/fcimb.2017.00177

**Published:** 2017-05-11

**Authors:** Muhammad A. Rasheed, Jingjing Qi, Xifang Zhu, He Chenfei, Harish Menghwar, Farhan A. Khan, Gang Zhao, Muhammad Zubair, Changmin Hu, Yingyu Chen, Huanchun Chen, Aizhen Guo

**Affiliations:** ^1^The State Key Laboratory of Agricultural Microbiology, Huazhong Agricultural UniversityWuhan, China; ^2^College of Veterinary Medicine, Huazhong Agricultural UniversityWuhan, China; ^3^Department of Biosciences, COMSATS Institute of Information TechnologySahiwal, Pakistan; ^4^Shanghai Veterinary Research Institute, Chinese Academy of Agricultural SciencesShanghai, China; ^5^Key Laboratory of Development of Veterinary Diagnostic Products, Ministry of Agriculture, Huazhong Agricultural UniversityWuhan, China; ^6^Hubei International Scientific and Technological Cooperation Base of Veterinary Epidemiology, Huazhong Agricultural UniversityWuhan, China

**Keywords:** attenuation, bioinformatics, genome, *Mycoplasma bovis*, virulence

## Abstract

*Mycoplasma bovis* is an important cause of bovine respiratory disease worldwide. To understand its virulence mechanisms, we sequenced three attenuated *M. bovis* strains, P115, P150, and P180, which were passaged *in vitro* 115, 150, and 180 times, respectively, and exhibited progressively decreasing virulence. Comparative genomics was performed among the wild-type *M. bovis* HB0801 (P1) strain and the P115, P150, and P180 strains, and one 14.2-kb deleted region covering 14 genes was detected in the passaged strains. Additionally, 46 non-sense single-nucleotide polymorphisms and indels were detected, which confirmed that more passages result in more mutations. A subsequent collective bioinformatics analysis of paralogs, metabolic pathways, protein-protein interactions, secretory proteins, functionally conserved domains, and virulence-related factors identified 11 genes that likely contributed to the increased attenuation in the passaged strains. These genes encode ascorbate-specific phosphotransferase system enzyme IIB and IIA components, enolase, L-lactate dehydrogenase, pyruvate kinase, glycerol, and multiple sugar ATP-binding cassette transporters, ATP binding proteins, NADH dehydrogenase, phosphate acetyltransferase, transketolase, and a variable surface protein. Fifteen genes were shown to be enriched in 15 metabolic pathways, and they included the aforementioned genes encoding pyruvate kinase, transketolase, enolase, and L-lactate dehydrogenase. Hydrogen peroxide (H_2_O_2_) production in *M. bovis* strains representing seven passages from P1 to P180 decreased progressively with increasing numbers of passages and increased attenuation. However, eight mutants specific to eight individual genes within the 14.2-kb deleted region did not exhibit altered H_2_O_2_ production. These results enrich the *M. bovis* genomics database, and they increase our understanding of the mechanisms underlying *M. bovis* virulence.

## Introduction

*Mycoplasma bovis* is a member of the *Mycoplasmataceae* family in the class of *Mollicutes*. It was first identified as a causative agent of bovine mastitis in 1961, and it was later recognized as an important pathogen of bovine respiratory disease in 1976 (Caswell and Archambault, 2007). Although it has been 56 years since *M. bovis* has been identified, there is limited understanding regarding its pathogenesis and virulence.

Compared with other bacteria, pathogenic *Mycoplasma* species have not been found to produce conventional toxins. Although ADP-ribosyl-transferase was preliminarily described as a possible toxin in a *Mycoplasma pneumoniae* strain that exhibits ADP-ribosyltransferase activity and elicits a distinct pattern of cytopathology in mammalian cells (Kannan and Baseman, 2006), it is difficult to distinguish pathogenic and non-pathogenic *Mollicutes* based on such virulence-related factors. Liproproteins and secretory proteins might contribute to bacterial virulence. Membrane lipoproteins, such as variable surface proteins (Vsps), enolase, and Vpmax, play significant roles in the adhesion of *M. bovis* to host cells (Burki et al., 2015). Subsequent invasion of host cells may be beneficial for *in vivo* survival and the dissemination of *M. bovis* to different sites in its hosts (Kleinschmidt et al., 2013). Regarding secretory proteins, only a few, including one *M. bovis* secretory protein, have been discovered (Zhang et al., 2016). Secondary metabolites such as hydrogen peroxide (H_2_O_2_) are considered to play a significant role in the pathogenesis of some *Mycoplasma* species, including *M. pneumoniae* (Hames et al., 2009) and *Mycoplasma mycoides* subsp. *mycoides* small colony (*MmmSC*) (Pilo et al., 2005). However, variations in H_2_O_2_ production might not correlate with *M. bovis* virulence (Schott et al., 2014). Instead, *M. bovis* might modulate the host immune response by suppressing interferon-γ and tumor necrosis factor-α production by invading immune cells to support its persistence and systemic dissemination (Mulongo et al., 2014). Genome sequences might provide more evidence that explains *Mycoplasma* pathogenesis at the genetic level. Currently, the genomes of 28 *M. bovis* strains, including the wild-type strain HB0801 and the three attenuated strains in the present study, have been sequenced and published (Li et al., 2011; Wise et al., 2011; Qi et al., 2012). Pathogenicity islands (PAIs) play a significant role in genome evolution and pathogenesis because many virulence-related factors are shared and acquired by PAIs. However, no PAIs and secretory systems have been detected in any *Mycoplasma* species (Guo and Wei, 2012). Using the virulence factors database (VFDB), some virulence genes were identified in the *M. bovis* genome (Parker et al., 2016), but their impact on *M. bovis* virulence remains to be investigated.

*M. bovis* was first isolated from the milk of a cow with mastitis in 1983 (Chen et al., 1983) and subsequently from lesioned lung tissue of a calf with pneumonia in 2008 in China (Qi et al., 2012). To develop candidate live vaccines against *M. bovis*, one strain, HB0801, which was isolated from lesioned lung tissue, was passaged continuously *in vitro*, and three strains that were passaged 115, 150, and 180 times, designated as *M. bovis* HB0801-P115, HB0801-P150, and HB0801-P180, respectively, were tested individually in cattle. The resulting clinical signs and pathological changes demonstrated that their virulence decreased with increasing numbers of passages (Zhang et al., 2014). Thus, a comparative genomics analysis of the virulent, wild-type strain HB0801 and these three attenuated strains might reveal some novel clues regarding the pathogenesis and virulence mechanisms of *M. bovis*.

Hence, in this study, we sequenced the complete genomes of these three attenuated strains and performed a comprehensive genomic analysis between the wild-type and the three attenuated strains. Based on the results, we hypothesize that a 14.2-kb deleted DNA fragment, single-nucleotide polymorphisms (SNPs), and indels probably affect the expression of some potential virulence-related proteins in the attenuated strains. In addition, the decreased capability to produce H_2_O_2_ in the attenuated strains was confirmed.

## Materials and methods

### *Mycoplasma* strains and culture conditions

*M. bovis* strain HB0801 (GenBank accession no. NC_018077.1) was isolated from the lung of infected beef cattle in Hubei Province, China, and its genome was fully sequenced by our laboratory (Qi et al., 2012). The *M. bovis* HB0801 attenuated strains HB0801-P115, HB0801-P150, and HB0801-P180, abbreviated as P115, P150, and P180, respectively, which exhibit progressively decreasing virulence, were used (Zhang et al., 2014). All the strains were propagated in pleuropneumonia-like organism (PPLO) medium supplemented with 10% horse serum (Thermo Fisher Scientific, Waltham, MA, USA) at 37°C for 48–72 h as described previously (Zhang et al., 2014).

### Library construction, DNA sequencing, and assembly

The DNA of strains P115, P150, and P180 was extracted using bacterial genomic DNA extraction kits (Tiangen, Beijing, China). The 454 pyrosequencing method was used to determine the whole genome sequences of strains P115, P150, and P180. Three paired-end sequencing libraries with 8-kb inserts were constructed at the China Tianjin Biochip Corporation (Tianjin, China). For each sample, one-fourth of a PicoTiterPlate was run on a Roche/454 GS FLX sequencer (454 Life Sciences, Branford, CT, USA) using titanium chemistry according to the manufacturer's recommendations. Finally, 83,750,973 bases with 286,408 reads were obtained for strain P115, while 80,961,239 bases with 277,020 reads were obtained for strain P150, and 83,589,289 bases with 260,056 reads were obtained for strain P180, resulting in 85.7-fold (P115), 82.8-fold (P150), and 85.5-fold (P180) depths of sequencing. All the reads for each genome were assembled *de novo* by the GS *De Novo* Assembler (version 2.6). Approximately 95% of reads were assembled for each genome, resulting in 11 scaffolds with 52 non-redundant contigs for strain P115, three scaffolds with 49 non-redundant contigs for strain P150, and one scaffold with 53 non-redundant contigs for strain P180. All the scaffolds were ordered and oriented according to the genome architecture of strain HB0801 (Qi et al., 2012). The N50 contig lengths of the large contigs (>1 kb) of each strain were 31,437 bp (P115), 31,456 bp (P150), and 31,042 bp (P180). The total numbers of base pairs of the non-redundant contigs were 916,922 bp (P115), 919,584 bp (P150), and 918,470 bp (P180), which agrees with the previously reported genome size of 991,702 bp for strain HB0801. To fill gaps within the scaffolds, polymerase chain reactions (PCRs) and capillary electrophoresis sequencing were performed with primers designed near the gaps. After gap filling, all the generated reads were respectively mapped to the corresponding full genome sequence using Burrows–Wheeler Aligner software (Li and Durbin, 2010). With the help of deep sequencing coverage, possible homopolymer errors resulting from the 454 sequencing method were rectified manually.

### Genome annotation and analysis

Open reading frames of the three passaged genomes (P115, P150, and P180) were predicted initially using Glimmer 3.02 (http://www.cbcb.umd.edu/software/glimmer/) and then modified using the translated nucleotide Basic Local Alignment Search Tool (BLAST) algorithm (http://blast.ncbi.nlm.nih.gov/) and compared to the genome of the primary strain HB0801 (GenBank accession no. CP002058). The functions of the coding sequences in the passaged strains were defined by referencing strain HB0801 (Qi et al., 2012). A comparative analysis between these four strains (P115, P150, P180, and HB0801) was conducted using MEGA 5.0 (Tamura et al., 2011) and Mauve 2.3.1 (Darling et al., 2004) genome alignment software. In addition, we detected differences in SNPs and indel sites among these strains.

### Confirmation of the 14.2-kb deleted region by PCR

To confirm the presence of the 14.2-kb deleted region during continuous passage, a putative lipoprotein-encoding gene (Mbov_0732) present in the deleted region was selected for PCR with forward (5′–AGCGACCAAAATACTAGAC –3′) and reverse (5′–TCGTTGCCACTGTATTCA–3′) primers using the following program: 95°C 3 min, followed by 30 cycles of 95°C for 30 s, 55°C for 30 s, and 72°C 2 min, followed by 72°C for 15 min and 16°C for 5 min.

### Confirmation of SNPs and indels

SNPs and indels might affect the expression of essential genes. Sixty-seven pairs of primers specific to the flanking sequences of the SNPs and indel sites were designed (Table S1) and Sanger DNA sequencing was performed with an ABI 3730 sequencer by China Tianjin Biochip Corporation (Tianjin, China).

### Bioinformatics analysis of predicted proteins

Differentially expressed proteins predicted by genome comparisons of the virulent HB0801 strain to its attenuated P115, P150, and P180 derivatives were classified into two categories: (i) proteins deleted in P115, P150, or P180 (Table 1) and (ii) proteins displaying non-sense SNPs and indels (Table 2). Protein functions and related metabolic pathways were assigned according to the Uniprot database (http://www.uniprot.org/) and the Kyoto Encyclopedia of Genes and Genomes (KEGG) database (http://www.kegg.jp/kegg-bin/show_organism?menu_type=pathway_maps&org=mbi), respectively. To determine whether proteins are secreted and contain a signal peptide, we used Pred-lipo (http://bioinformatics.biol.uoa.gr/PRED-LIPO/input.jsp), the SignalP 4.1 server for the prediction of classical secreted proteins (http://www.cbs.dtu.dk/services/SignalP/), and the SecretomeP 2.0 server for the prediction of non-classical secreted proteins (http://www.cbs.dtu.dk/services/SecretomeP/). Moreover, to assess whether the SNPs were present at active sites or domains of the proteins and to obtain the conserved domains in the secretory proteins, we used the National Center for Biotechnology Information (NCBI) conserved domain database (http://www.ncbi.nlm.nih.gov/Structure/cdd/wrpsb.cgi). Paralogs of the genes and percentages of identity between paralogs were determined with the NCBI BLAST algorithm (http://blast.ncbi.nlm.nih.gov/Blast.cgi). Protein–Protein interactions were obtained using the STRING database (www.string-db.org).

**Table 1:** **The 14 genes present in the 14.2-kb deleted region of the three attenuated strains**.

**Gene number**	**Start site**	**Stop site**	**Encoding proteins**
Mbov_0722	854,329	854,446	Ascorbate-specific PTS system enzyme IIB component
Mbov_0723	854,545	856,353	Ascorbate-specific PTS system enzyme IIA component
Mbov_0724	856,405	857,466	Phosphotriesterase family protein
Mbov_0725	857,812	858,669	Predicted hydrolases of the HAD superfamily
Mbov_0726	858,857	859,093	Hypothetical protein
Mbov_0727	859,172	860,956	DNA methyltransferase
Mbov_0728	861,333	861,512	Amino-terminal fragment of hypothetical protein; pseudo
Mbov_0729	861,695	862,609	Putative lipoprotein
Mbov_0730	862,734	863,949	Lipoprotein containing a frameshift mutation; pseudo
Mbov_0732	864,069	865,058	Putative lipoprotein
Mbov_0733	865,436	865,627	Hypothetical protein
Mbov_0734	865,790	866,689	Conserved hypothetical protein
Mbov_0735	866,753	868,621	Type III RM system methylase; pseudo
Mbov_0856	865220	865,383	Putative lipoprotein

**Table 2:** **SNPs and indels in the three attenuated strains, compared with strain HB0801, after resequencing and PCR confirmation**.

**Serial number**	**Gene number**	**P115**	**P150**	**P180**	**SNP site**	**Encoding protein**
1	Mbov_0018	C-A	C-A	C-A	S17,328	Simple sugar ABC transporter ATP-binding protein
2	Mbov_0134	C-A	C-A	C-A	S151,187	Spermidine/putrescine ABC transporter ATP-binding protein
3	Mbov_0160	C-T	C-T	C-T	S183,285	D-lactate dehydrogenase
4	Mbov_0206	C-T	C-T	C-T	S241,786	Ribose-phosphate pyrophosphokinase
5	Mbov_0350	C-A	C-A	C-A	S418,864	Putative lipoprotein
6	Mbov_0482	T-C	T-C	T-C	S562,835	Enolase
7	Mbov_0482	G-A	G-A	G-A	S562,695	Enolase
8	Mbov_0518	C-T	C-T	C-T	S609,729	Putative lipoprotein
9	Mbov_0533	C-T	C-T	C-T	S628,787	Cation transporting ATPase
10	Mbov_0567	C-A	C-A	C-A	S669,593	Phosphate acetyltransferase
11	Mbov_0584	C-A	C-A	C-A	S691,444	Putative transmembrane protein
12	Mbov_0640	C-A	C-A	C-A	S742,189	Large-conductance mechanosensitive ion channel
13	Mbov_0714	C-A	C-A	C-A	S848,007	Predicted integral membrane protein
14	Mbov_0767	G-C	G-C	G-C	S901,268	Conserved hypothetical protein
15	Mbov_0155	–	C-T	C-T	S175,797	Pyruvate kinase
16	Mbov_0165	–	C-T	C-T	S187,262	Hypothetical protein
17	Mbov_0522	–	C-T	C-T	S618,377	Phosphopentomutase
18	Mbov_0579	–	A-T	A-T	S684,890	Membrane lipoprotein P81
19	Mbov_0641	–	G-A	G-A	S743,651	Heat shock protein GrpE
20	Mbov_0714	–	C-A	C-A	S845,529	Predicted integral membrane protein
21	Mbov_0797	–	G-A	G-A	S929,799	VspHB0801-5
22	Mbov_0832	–	A-C	A-C	S964,804	Thioredoxin
23	Mbov_0049	–	G-A	–	S58,153	Putative lipoprotein
24	Mbov_0111	C-T	–	–	S120,789	Putative lipoprotein
25	Mbov_0212	C-T	–	–	S247,443	Transketolase
26	Mbov_0248	G-T	–	–	S284,709	Integrase
27	Mbov_0299	–	–	G-A	S350,175	NADH dehydrogenase
28	Mbov_0328	–	–	C-A	S388,007	Exopolyphosphatase-related protein
29	Mbov_0393	C-A	–	–	S457,329	Putative membrane lipoprotein (ICEB-1 encoded)
30	Mbov_0403	C-T	–	–	S472,982	Transcription elongation factor
31	Mbov_0540	–	–	G-A	S638,676	Putative transmembrane protein
32	Mbov_0553	–	–	C-A	S628,101	Cation transporting ATPase
33	Mbov_0565	–	–	C-T	S667,404	L-lactate dehydrogenase
34	Mbov_0702	–	–	G-A	S833,489	Transcriptional accessory protein
35	Mbov_0738	–	–	T-C	S871,124	Putative transmembrane protein
36	Mbov_0742	–	–	G-A	S876,089	Glycerol ABC transporter ATP binding component
37	Mbov_0816	–	–	G-A	S950,221	Hypothetical protein
38	Mbov_0824	–	T-C	–	S957,308	Peptide chain release factor
1	Mbov_0338	–	Del A	Del A	D399,903	Alcohol dehydrogenase
2	Mbov_0340	–	Del CTAGT	Del CTAGT	D402,407	Putative transmembrane protein
3	Mbov_0347	–	Del CT	Del CT	D414,907	Putative lipoprotein
4	Mbov_0356	Del C	–	Del C	D423,697	Pseudogene of cytosine-specific methyltransferase
5	Mbov_0525	–	Del G	Del G	D620,722	Putative membrane lipoprotein
6	Mbov_0581	–	–	Del A	D687,694	Multiple sugar ABC transporter ATP-binding protein
7	Mbov_0656	Del G	Del G	Del G	D768,061	Putative lipoprotein (variable)
8	Mbov_0682	–	Del CT	Del CT	D798,998	Putative lipoprotein

*S, SNP; Del, deletion. The data are categorized into four categories. 1, SNP present in all three passages; 2, SNP present in two passages; 3, SNP present in one passage; 4, deletions found in the genes*.

The involvement of these proteins in virulence was further confirmed based on the whole genome of *M. bovis* HB0801 using the VFDB (http://www.mgc.ac.cn/VFs/) (Chen et al., 2016). The amino acid sequences of all the proteins were obtained from the NCBI. Each protein was aligned individually against the VFDB full dataset by the BLAST algorithm. A matrix was created by VFDB output, including VFDB hits against each protein in *M. bovis*, a related BLAST score, and an E-value. The matrix was filtered based on a BLAST score ≥80.

### H_2_O_2_ production assay for representative passaged strains and mutants

H_2_O_2_ production was measured as described previously in *Mycoplasma mycoides* subsp. *capri* (Allam et al., 2012). Briefly, eight transposon-disrupted mutants in the deleted region of the attenuated strains, including Mbov_0723, Mbov_0724, Mbov_0725, Mbov_0727, Mbov_0730, Mbov_0732, Mbov_0734, and Mbov_0735, as well as different passages of *M. bovis* HB0801, including P1, P25, P50, P75, P100, P115, P150, and P180, were grown to mid-logarithmic phase. The transposon-disrupted mutants were prepared using the wild-type HB0801 strain and a Tn4001 transposon. The transposon disrupted the abovementioned genes that are present in the deleted region mentioned in Table 1. Moreover, the Mbov_0723, Mbov_0724, Mbov_0725, Mbov_0727, Mbov_0730, Mbov_0732, Mbov_0734, and Mbov_0735 genes were transposon-disrupted at positions 85,6071, 85,7345, 858,521, 860,287, 863,914, 864,069, 866,042, and 866,786, respectively, in the genome. To assess H_2_O_2_ production, bacteria were harvested by centrifugation at 15,400 g for 4 min at 4°C. The pellets were washed three times in incubation buffer (67.7 mM HEPES, pH 7.3; 140 mM NaCl, and 7 mM MgCl_2_). After the final wash, the *M. bovis* pellets were resuspended in incubation buffer to a density of 10^8^ cells/ml and incubated for 20 min at 37°C. After the incubation, 10 μl of glycerol (10 mM) was added, and the samples were kept at 37°C for 30 min. H_2_O_2_ production was determined using commercial H_2_O_2_ assay kits (Cayman Chemical, Ann Arbor, MI, USA). Standard curve determination and specificity quality controls were performed according to the instructional manual. The experiments were performed in three independent replicates.

### Statistical analysis

To determine the statistical significance among the differences in SNPs after comparing all the genes of *M. bovis*, as well as among different SNP and indel types, a *z*-test for proportions (*z* = *p*ˆ-*p*0/√*p*0(1 − *p*0)/*n*) was applied to generate a *p*-value. Moreover, to determine the statistical significance of the genes involved in different KEGG pathways, a KEGG enrichment analysis was performed using the KOBAS 2.0 web server, and Fisher's exact test was used to analyze the data (Xie et al., 2011). To analyze differences in H_2_O_2_ production among the different strains, two-way analysis of variance was performed using GraphPad Prism version 5.0 (GraphPad Software, San Diego, CA, USA). *p* < 0.05 were considered to be significantly different and are marked with an asterisk in the figures, while *p* < 0.01 were considered to very significantly different and are marked with two asterisks in the figures.

### Nucleotide sequence accession numbers

The complete genome sequences of the three attenuated strains were deposited in the GenBank database with the accession numbers CP007589 for P115, CP007590 for P150, and CP007591 for P180.

## Results and discussion

### A 14.2-kb deleted fragment is present in the genomes of the three attenuated strains

**Figure 1:**
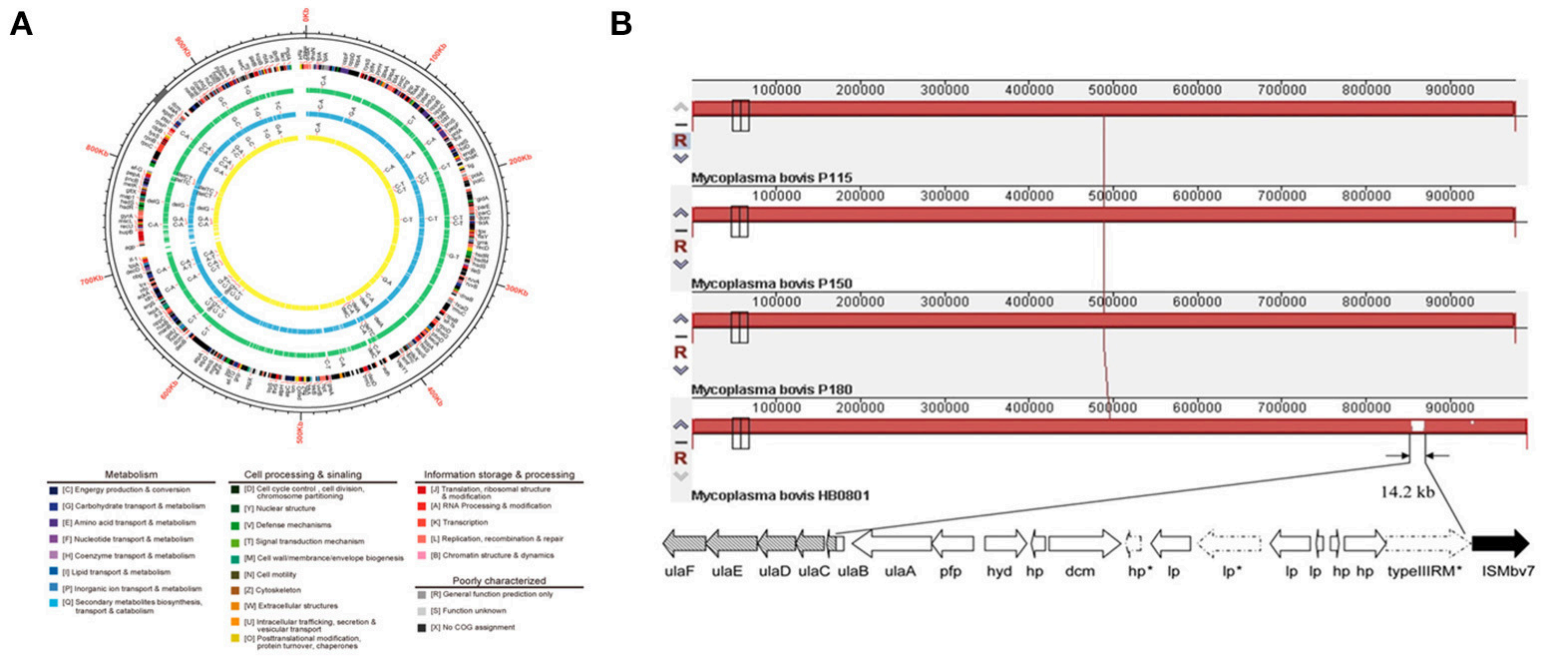
**Genomic comparison of ***M. bovis*** strains HB0801 (P1), P115, P150, and P180. (A)** Genome structures of the *M. bovis* HB0801 strain and the three attenuated strains; **(B)** Serial propagation of HB0801 resulted in the deletion of a 14.2-kb fragment in the genome of the three attenuated strains. The red lines show the lengths of the genomes of respective strains. The lengths of the P115, P150, and P180 strains are reduced and shown in dashes at the ends of the lines.

Because of a lack of genetic tools and limited research techniques, there is limited understanding of the pathogenesis and virulence-related factors of *M. bovis*. Hence, the whole genomes of strains P115, P150, and P180 were sequenced and assembled, and a comparative study was performed to identify significant virulence-related factors. The genome structures of the virulent *M. bovis* HB0801 strain and the three attenuated strains are shown in Figure 1A. An analysis of the three attenuated strains and the wild-type HB0801 strain revealed that all the genomes of strains P115, P150, and P180 lost a 14.2-kb fragment compared with strain HB0801 (Figure 1B and Table 1). Further analysis of the deleted fragment showed that it consists of 14 putative genes encoding ascorbate-specific phosphotransferase system (PTS) system enzyme IIB and IIA components, a phosphotriesterase family protein, predicted hydrolases of the haloacid dehalogenase (HAD) superfamily, DNA methyltransferase, and a type III restriction-modification (RM) system methylase, as shown in Table 1. In addition, 38 strains that were passage between one and 115 times were shown by PCR to have a deletion in the Mbov_0732 gene. The results demonstrated that the 14.2-kb deletion occurred in passage 115 and onward as shown in Figure S1, indicating that the deletion was stably maintained in the passaged strains after its occurrence.

### SNPs and indels

In addition to the 14.2-kb deleted region, a genome comparison between strain HB0801 and the attenuated strains revealed many SNPs and indels. Sixty-seven non-sense SNP sites were tested with PCR and DNA sequencing, and 38 SNPs were confirmed (Table 2). In agreement with the increasing attenuation tendency resulting from continuous passage, strains that were subjected to more passages had more SNPs, and the SNPs in the strains that were subjected to fewer passages were shared by the higher passaged strains. The numbers of SNPs were 31, 24, and 19 for the P180, P150, and P115 strains, respectively. In addition, eight nucleotide deletions were found, and their frequencies of occurrence followed a pattern that was similar to that of the SNPs (Table 2). These changes in the genomes were analyzed statistically with a *z*-test of proportions, and the results showed that there were significant differences in the numbers of SNPs, indels, and SNPs+indels between the wild-type strain and each of the attenuated strains, and between any two attenuated strains (normalized *p* < 0.00001).

### Detection of paralogs in the *M. bovis* genome

Theoretically, the functions of some genes containing SNPs or deletions could be compensated by their paralogs in the genome. Hence, the FASTA sequences of such paralogs were aligned with a reference protein by the BLAST algorithm to determine the percentage of identity between them (Table S2); a reference protein is a protein that is found in our genomics data (Tables 1, 2). The results showed that the proteins that have paralogs in our genomics data included four of the 14 genes in the 14.2-kb deleted region (Mbov_0727, Mbov_0729, Mbov_0732, and Mbov_0856) and 14 of the 46 genes containing SNPs or indels (Mbov_0018, Mbov_0049, Mbov_0111, Mbov_0134, Mbov_0338, Mbov_0347, Mbov_0350, Mbov_0393, Mbov_0518, Mbov_0525, Mbov_0581, Mbov_0682, Mbov_0742, and Mbov_0832). Hence, the genes responsible for virulence attenuation would likely include the other 10 genes in the 14.2-kb deleted region and the 32 genes with SNPs and indels, but which lacked paralogs.

### Prediction of protein–protein interactions

**Figure 2:**
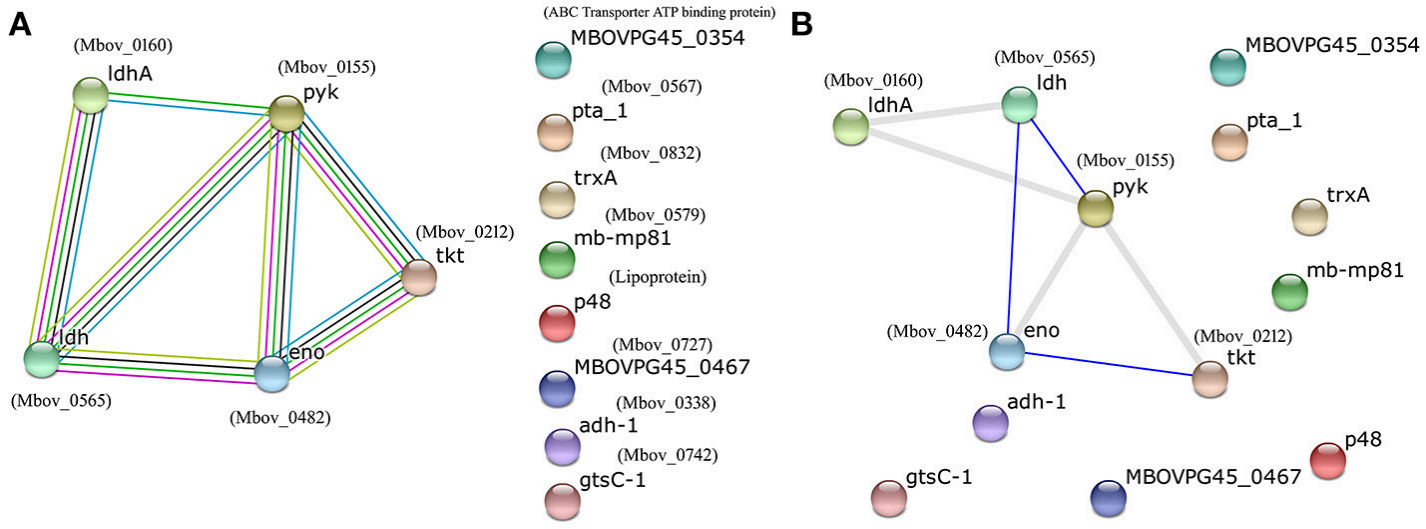
**Protein–protein interaction map of the available proteins from the list of analyzed proteins from Tables 1, 2**. Different colored lines show different types of interactions. Hence, more lines show more interactions and an increased probability of a protein–protein interaction. The image and related details were retrieved from the STRING database (http://www.string-db.org/). **(A)** Evidence view; **(B)** Interactive view. In evidence view, all possible interactions are shown. Different colored lines show different types of interactions e.g., gene fusion, co-occurrence, co-expression, experiments, databases, text mining etc. While in interactive view, the interactive proteins are clustered together based on interaction and co-occurrence.

The proteins listed in Tables 1, 2 were input into the STRING database to predict potential protein-protein interactions using the type strain *M. bovis* PG45 as the database default reference. Protein–protein interaction maps are given for 13 of the 60 input proteins (Figure 2). Different colored lines show different types of interactions. Hence, more lines show more interactions and an increased probability of a protein-protein interaction. The interactions among the proteins are highlighted in evidence view (Figure 2A). In evidence view, all possible interactions are shown. Different colored lines show different types of interactions e.g., gene fusion, co-occurrence, co-expression, experiments, databases, text mining etc. While in interactive view, the interactive proteins are clustered together based on interaction and co-occurrence. The evidence (Figure 2A) and interactive views (Figure 2B) showed that the most significant proteins that are likely to interact include enolase (Mbov_0482), pyruvate kinase (Mbov_0155), transketolase (Mbov_0212), L-lactate dehydrogenases (Mbov_0565), and D-lactate dehydrogenases (Mbov_0160), suggesting that these proteins might function together, and thus, that they are the strongest candidates for explaining the attenuated virulence of highly passage *M. bovis* strains.

### Pathway enrichment assay

**Figure 3:**
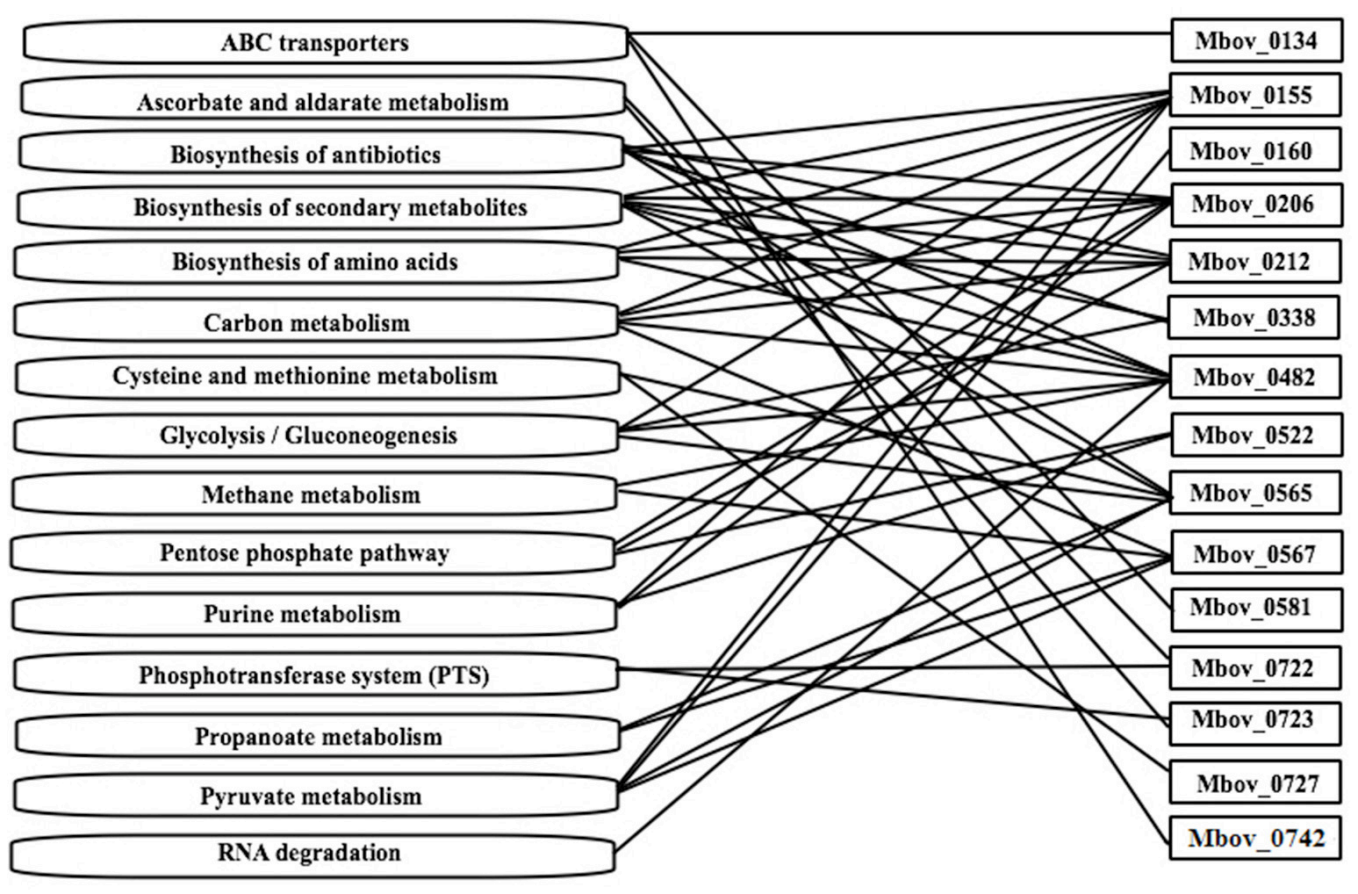
**The mutated genes from Tables 1, 2 that are enriched in some metabolic pathways**. Fifteen genes were shown to be enriched in 15 metabolic pathways. Ten of the fifteen genes are involved in more than one pathway.

A KEGG enrichment analysis was performed to determine whether the mutated genes in Tables 1, 2 are enriched in some important metabolic pathways. Fifteen genes were shown to be enriched in 15 metabolic pathways. Furthermore, 10 of the 15 genes were involved in more than one pathway (Figure 3 and Table S3). The 10 genes and the involved numbers of pathways numbers are Mbov_0155 and Mbov_0482 (seven), Mbov_0206 and Mbov_0565 (six), Mbov_0212 (five), Mbov_0567 (four), Mbov_0338 (three), and Mbov_0522, Mbov_0722, and Mbov_0723 (two). Among them, Mbov_0155, Mbov_0212, Mbov_0482, and Mbov_0565 were also identified in the protein-protein interaction analysis. Theoretically, proteins involved in more pathways should be more likely to play a significant role in *M. bovis* virulence. However, among the pathways, biosynthesis of secondary metabolites, biosynthesis of antibiotics, carbon metabolism, pyruvate metabolism, biosynthesis of amino acids, glycolysis/gluconeogenesis, pentose phosphate pathway, purine metabolism, and ATP-binding cassette (ABC) transporters were enriched in more pathways (six, six, five, four, four, four, three, three, and three, respectively).

### Conserved domain analysis

Twelve predicted secretory proteins with signal peptides, which includes those encoded by 10 genes (Mbov_0049, Mbov_0111, Mbov_0347, Mbov_0350, Mbov_0393, Mbov_0518, Mbov_0525, Mbov_0579, Mbov_0682, and Mbov_0797) that have SNPs (Table 2) and the Mbov_0729 and Mbov_0856 genes, which are deleted, were identified (Table 1). A conserved domain analysis (**Table 4**) identified the presence of conserved domains in the eight secretory proteins mentioned in Table 3, but not in Mbov_0111, Mbov_0393, Mbov_0525, and Mbov_0856. Liproproteins and secretory proteins might contribute to bacterial virulence and pathogenesis because they are involved in many phenomena, ranging from cellular physiology through immune responses to virulence. Some secretory proteins were discovered previously in *M. bovis* (Khan et al., 2016; Zhang et al., 2016). Many other genes in the *M. bovis* genome are predicted to encode putative lipoproteins and secretory proteins, but whether these proteins exist in a functional form and how they are related to virulence need to be determined. The most significant conserved domains with available functions include CDC45 (Mbov_0797), DUF1388 (Mbov_0797), structural maintenance of chromosomes (SMC) (Mbov_0049), Rad23 (Mbov_0682), and peptidase C19 (Mbov_0729) (Table 4). CDC45 is required for the initiation of DNA replication (Saha et al., 1998). Members of the DUF1388 family function as the main targets for neurofilament-directed protein kinases *in vivo*. SMC proteins are essential for successful chromosome transmission during replication, and segregation of the genome, including chromosome condensation, recombination, DNA repair, and epigenetic silencing of gene expression (Haering et al., 2002). Rad23 family proteins are used for targeting nucleotide excision repair to specific parts of the genome. Peptidase C19 is a deubiquitinating enzyme that can deconjugate ubiquitin or ubiquitin-like proteins from ubiquitin-conjugated proteins (De Jong et al., 2006). The functions of the other domains are currently unknown.

**Table 3:** **Putative secretory proteins with signal peptides**.

**Gene**	**Name**	**D**	**Smean**	**Cmax**	**Pos**
Mbov_0049	Putative lipoprotein	0.638	0.724	0.556	33
Mbov_0111	Putative lipoprotein	0.498	0.716	0.181	26
Mbov_0347	Putative lipoprotein	0.623	0.798	0.363	32
Mbov_0350	Putative lipoprotein	0.566	0.845	0.184	32
Mbov_0393	Putative membrane lipoprotein (ICEB-1 encoded)	0.465	0.68	0.185	24
Mbov_0518	Putative lipoprotein	0.551	0.88	0.176	32
Mbov_0525	Putative membrane lipoprotein	0.693	0.893	0.344	23
Mbov_0579	Membrane lipoprotein P81	0.591	0.884	0.184	25
Mbov_0682	Putative lipoprotein	0.722	0.853	0.533	32
Mbov_0729	Putative lipoprotein	0.568	0.792	0.206	25
Mbov_0797	VspHB0801-5	0.659	0.946	0.238	23
Mbov_0856	Putative lipoprotein	0.641	0.918	0.229	25

*Secretory proteins with signal peptides were identified by Pred-lipo (http://bioinformatics.biol.uoa.gr/PRED-LIPO/input.jsp), the SignalP 4.1 server for the prediction of classically secreted proteins (http://www.cbs.dtu.dk/services/SignalP/), and the SecretomeP 2.0 server for the prediction of non-classically secreted proteins (http://www.cbs.dtu.dk/services/SecretomeP/) using a default cutoff value of 0.45. Cmax is the signal peptide cleavage site score. Smean is the average score of the putative signal peptide. D is the discrimination score that differentiates signal peptides from non-signal peptides. Pos is the cleavage position of the protein*.

**Table 4:** **Conserved domains in the secretory proteins**.

**Gene**	**Name**	**Domain**	**Name**
Mbov_0049	Putative lipoprotein	330–636	SMC_prok_A
Mbov_0347	Putative lipoprotein	29–176	PLN02967
Mbov_0350	Putative lipoprotein	304–660	DUF31
Mbov_0518	Putative lipoprotein	392–764	DUF31
Mbov_0579	Membrane lipoprotein P81	257–482 & 570–698	Lipoprotein_X & Lipoprotein_10
Mbov_0682	Putative lipoprotein	6–175, 183–253	PRK08581, rad23
Mbov_0729	Putative lipoprotein	189–286	Peptidase C19
Mbov_0797	VspHB0801-5	46–74, 70–98, 195–253, 152–180	DUF1388, DUF1388, CDC45, DUF1388

*The analysis highlights the presence of conserved domains in the secretory proteins. The analysis was performed using the NCBI server (http://www.ncbi.nlm.nih.gov/Structure/cdd/wrpsb.cgi)*.

### The virulence-related factors identified by the VFDB

To further investigate the virulence-related factors that contribute to the attenuation of highly passaged *M. bovis* strains, all the proteins of *M. bovis* were analyzed using the VFDB. In the VFDB full dataset, all proteins related to known and predicted virulence-related factors are present. Seventy-two genes in the *M. bovis* genome were shown to encode virulence-related factors based on a BLAST score ≥80 (Table 5). Although no virulence-related factor-encoding genes were found in the 14.2-kb deleted region, eight genes overlapped with those containing SNPs and indels (Table 2). In decreasing order of BLAST scores, they are Mbov_0482, Mbov_0533, Mbov_0581, Mbov_0338, Mbov_0134, Mbov_0742, Mbov_0018, and Mbov_0797.

**Table 5:** **Virulence-related factor identification in ***M. bovis*** using the VFDB**.

**Gene id**	**VFDB hit**	**Score**	**E-value**
Mbov_0560	IS1634AV transposase [*MmmSC*]	1,060	0
Mbov_0481	elongation factor Tu [*Mycoplasma agalactiae*]	754	0
Mbov_0016	predicted lipoprotein [monocytic differentiation factor] [*M. agalactiae*]	743	0
Mbov_0842	hypothetical protein [Mg^2+^ transport] [*Salmonella enterica* subsp. *arizonae*]	657	0
Mbov_0174	P48, predicted lipoprotein [*M. agalactiae*]	627	1.00E-180
Mbov_0103	pyruvate dehydrogenase E1 component [*M. agalactiae*]	611	1.00E-175
Mbov_0703	endopeptidase Clp ATP-binding chain C [*Listeria monocytogenes*]	546	1.00E-155
Mbov_0157	molecular chaperone DnaK [*Chlamydia trachomatis*]	475	1.00E-134
Mbov_0482	phosphopyruvate hydratase [*Streptococcus agalactiae*]	443	1.00E-124
Mbov_0033	oligopeptide ABC transporter [*M. mycoides* subsp. *mycoides*]	414	1.00E-116
Mbov_0062	glyceraldehyde 3-phosphate dehydrogenase [*Streptococcus sanguinis*]	404	1.00E-113
Mbov_0310	SecA DEAD domain protein [*Mycobacterium vanbaalenii*]	392	1.00E-109
Mbov_0520	NAD dependent DNA ligase [*Leptospira interrogans* serovar Lai]	332	3.00E-91
Mbov_0428	PgPepO oligopeptidase [*Mycobacterium* sp. JLS]	280	3.00E-75
Mbov_0488	peptide methionine sulfoxide reductase [*Neisseria meningitidis*]	280	8.00E-76
Mbov_0687	lipid A ABC exporter [*Haemophilus somnus*]	273	2.00E-73
Mbov_0302	RNA polymerase, RpoD family [*Mycobacterium gilvum*]	236	3.00E-62
Mbov_0491	lipid A export ATP-binding protein MsbA [*Haemophilus influenzae* PittGG]	234	1.00E-61
Mbov_0068	lipoyltransferase and lipoate-protein ligase family protein [*L. monocytogenes*]	231	3.00E-61
Mbov_0010	putative lipase protein ligase A [*Listeria ivanovii* subsp. *ivanovii*]	226	1.00E-59
Mbov_0009	hypothetical protein [*Listeria innocua*]	225	4.00E-59
Mbov_0533	magnesium-translocating P-type ATPase [*S. enterica* subsp. *enterica*]	222	7.00E-58
Mbov_0280	predicted lipoprotein [*M. agalactiae*]	207	6.00E-54
Mbov_0693	P65 lipoprotein-like protein [*Mycoplasma mobile* 163K]	201	7.00E-51
Mbov_0539	probable phosphomannomutase [*Haemophilus ducreyi*]	199	5.00E-51
Mbov_0532	glucose-1-phosphate uridylyltransferase [*Bacillus thuringiensis*]	197	5.00E-51
Mbov_0490	lipid transporter ATP-binding/permease [*H. influenzae*]	187	2.00E-47
Mbov_0675	adenosine synthase A [*Streptococcus pyogenes*]	177	1.00E-44
Mbov_0674	membrane nuclease [*Mycoplasma pulmonis*]	173	2.00E-43
Mbov_0796	variable surface lipoprotein W [*M. agalactiae* PG2]	168	3.00E-42
Mbov_0438	type III secretion system ATPase [*Aeromonas salmonicida* subsp. *salmonicida*]	160	2.00E-39
Mbov_0798	variable surface lipoprotein V [*M. agalactiae* PG2]	160	8.00E-40
Mbov_0688	fused lipid transporter subunits of ABC superfamily [*H. influenzae*]	159	4.00E-39
Mbov_0581	ABC transporter ATP-binding protein [*Mycobacterium leprae*]	157	2.00E-38
Mbov_0341	P65 lipoprotein-like protein [*M. mobile*]	154	9.00E-37
Mbov_0440	type III secretion system ATPase [*Desulfovibrio vulgaris*]	153	3.00E-37
Mbov_0745	(CbuG_0446) hypothetical protein [type IV secretion system effector] [*Coxiella burnetii* CbuG_Q212]	153	8.00E-38
Mbov_0115	oligopeptide ABC transporter [*MmmSC*]	150	4.00E-36
Mbov_0338	alcohol dehydrogenase [MymA operon] [*Mycobacterium* sp. JLS]	140	2.00E-33
Mbov_0353	zinc-type alcohol dehydrogenase [*Mycobacterium ulcerans*]	134	1.00E-31
Mbov_0508	flagellum-specific ATP synthase FliI [*Legionella pneumophila* subsp. *pneumophila*]	131	1.00E-30
Mbov_0038	predicted cytoskeletal protein [*Mycoplasma penetrans*]	124	1.00E-27
Mbov_0134	maltodextrin import ATP-binding protein [*Mycobacterium abscessus* subsp. *bolletii*]	122	4.00E-28
Mbov_0168	trigger factor [*Streptococcus mutans*]	120	2.00E-27
Mbov_0595	hemolysin secretion protein HlyB [*Escherichia coli*]	120	8.00E-28
Mbov_0742	sugar ABC transporter ATP-binding protein [*M. gilvum* Spyr1]	117	1.00E-26
Mbov_0034	oligopeptide ABC transporter [*M. mycoides* subsp. *mycoides*]	113	3.00E-25
Mbov_0535	phthiocerol dimycocerosate and phenolic glycolipid biosynthesis and transport [*Mycobacterium avium* subsp. *paratuberculosis*]	110	9.00E-25
Mbov_0509	HrcN [type III secretion system] [*Pantoea stewartii* subsp. *stewartii* str. SS104]	106	4.00E-23
Mbov_0843	HitC iron(III) ABC transporter ATP-binding protein [*H. influenzae*]	104	1.00E-22
Mbov_0099	lipoprotein diacylglyceryl transferase [*L. innocua*]	103	1.00E-22
Mbov_0233	serine/threonine protein kinase [*Mycobacterium tuberculosis*]	102	1.00E-27
Mbov_0291	fibronectin-binding protein [*Tannerella forsythia*]	100	5.00E-21
Mbov_0810	segregation and condensation protein B [*S. agalactiae*]	100	5.00E-22
Mbov_0029	ABC transporter, ATP-binding protein [*H. somnus*]	99	3E-21
Mbov_0594	iron ABC transporter ATP-binding protein [*Corynebacterium pseudotuberculosis*]	96	3.00E-20
Mbov_0018	iron-uptake permeate ATP-binding protein [*Neisseria lactamica*]	93	4.00E-19
Mbov_0152	lipopolysaccharide core biosynthesis protein [*Helicobacter pylori*]	92	8.00E-20
Mbov_0114	oligopeptide ABC transporter [*MmmSC*]	89	6.00E-18
Mbov_0121	ABC-type transporter [*Enterococcus faecalis*]	89	9.00E-18
Mbov_0375	Putative short-chain type dehydrogenase/reductase [*Mycobacterium canettii*]	89	3.00E-18
Mbov_0554	LicA protein [LOS] [*H. somnus* 129PT]	89	3.00E-18
Mbov_0312	AdhD alcohol dehydrogenase [*Mycobacterium intracellulare* MOTT-02]	88	7.00E-18
Mbov_0279	HlyC/CorC family [hemolysin] [*Clostridium botulinum* A str. ATCC 19397]	87	3.00E-17
Mbov_0232	protein phosphatase PrpC [*L. monocytogenes*]	86	2.00E-17
Mbov_0064	beta-1,3 galactosyltransferase [*Campylobacter jejuni* subsp. *jejuni*]	85	6.00E-17
Mbov_0784	elongation factor Tu [EF-Tu] [*M. pulmonis* UAB CTIP]	84	3.00E-16
Mbov_0427	Dot/Icm type IV secretion system effector [*L. pneumophila*]	83	1.00E-16
Mbov_0797	variable surface lipoprotein W (VpmaW precursor) [*M. agalactiae*]	82	6.00E-16
Mbov_0156	variable surface lipoprotein Y [*M. agalactiae*]	80	2.00E-15
Mbov_0307	iron-dicitrate transporter ATP-binding subunit [*Vibrio parahaemolyticus*]	80	1.00E-15
Mbov_0845	ATPase [Proteasome-associated proteins] [*Mycobacterium marinum*]	80	4.00E-15

*Virulence proteins found in the VDFB for M. bovis HB0801 proteins using BLAST are listed as a VFDB hit. Scores and E-values were generated by the BLAST algorithm. The data were filtered by BLAST scores ≥ 80*.

### Analysis of critical deleted genes and genes with SNPs and indels

By combining the above results for the deleted genes and the genes with SNPs and indels, 11 critical genes that likely contribute to the attenuation of highly passaged *M. bovis* strains were identified, and they include (in order of decreasing importance) Mbov_0722, Mbov_0723, Mbov_0482, Mbov_0565, Mbov_0155, Mbov_0581, Mbov_0742, Mbov_0299, Mbov_0212, Mbov_0797, and Mbov_0567.

Among the deleted genes, Mbov_0722 and Mbov_0723 are highlighted. They encode ascorbate-specific PTS enzyme IIB and IIA components, respectively, which function in ascorbate and aldarate metabolism pathways by involving the PTS, the major carbohydrate transport system in bacteria, and they are responsible for the conversion of L-ascorbate into L-ascorbate-6 phosphate, as shown in Figure S2 (Postma et al., 1993). The final product of this pathway is D-xylulose-5 phosphate, which is an intermediate in the pentose phosphate pathway. As is known, the primary purpose of this pathway is to generate a reducing equivalent of NADPH that can be used in reductive biosynthesis reactions, while the production of the pentose phosphate pathway intermediates ribose 5-phosphate and erythrose 4-phosphate are used to synthesize nucleotides and nucleic acids, and aromatic amino acids, respectively. In addition, this sugar has a role in gene expression, mainly by promoting the ChREBP transcription factor in the well-fed state (Iizuka and Horikawa, 2008). Hence, because of the deficiencies of both proteins in the attenuated strains, these related metabolic functions would be less efficient. Because the occurrence of the 14.2-kb deleted region began at passage 115 and was maintained from passages 115 through 180, these metabolic defects might contribute to the increasing attenuation of highly passaged *M. bovis* strains.

The enolase encoded by Mbov_0482 is considered to be a virulence-related factor, and it participates in seven metabolic pathways and interacts with other proteins as described previously. It catalyzes the reversible conversion of 2-phosphoglycerate into phosphoenolpyruvate. This reaction is present in the glycolysis/gluconeogenesis pathways of *M. bovis*, as shown in Figure S3. Prokaryotic α-enolase may contribute to pathophysiological processes (Pancholi, 2001). A surface-associated enolase is an adhesion-related factor of *M. bovis* that contributes to adherence by binding plasminogen (Song et al., 2012). The immunogenicity of enolase has also been observed in *Mycoplasma synoviae* (Bercic et al., 2008) and *Mycoplasma capricolum* subsp. *capripneumoniae* (Zhao et al., 2012). Hence, enolase might be a significant protein that contributes to the virulence of *M. bovis*. However, two SNPs were not detected inside the enolase-encoding gene, but were located approximately 300 bp upstream of the gene, suggesting that both SNPs might cause some change in the promoter region.

Like enolase, L-lactate dehydrogenase encoded by Mbov_0565 is another protein that is important for metabolism and as a virulence-related factor. It interconverts L-lactate to pyruvate. Additionally, this enzyme works in many metabolic pathways, including glycolysis/gluconeogenesis, cysteine and methionine metabolism, pyruvate metabolism, and propanoate metabolism. Hence, this lactate dehydrogenase plays significant roles in a variety of metabolic processes. Moreover, L-lactate dehydrogenase was shown to be surface expressed and to interact with plasminogen (Grundel et al., 2015). Furthermore, because of the SNP in the L-lactate dehydrogenase-encoding gene, a polar amino acid (threonine) was converted to a non-polar amino acid (methionine).

Pyruvate kinase (Mbov_0155) is also a very active protein in metabolism and energy production. It catalyzes the conversion of phosphoenolpyruvate to pyruvate (Figure S4). Then, mycoplasmas generate ATP via a proton-translocating ATP synthase by oxidizing organic acids (pyruvate and lactate) to acetate and CO_2_. Moreover, the pyruvate kinase present in the genome of *Mycoplasma suis* is proposed to be required for the conversion of all NDPs and dNDPs to NTPs and dNTPs, respectively (Pollack et al., 2002). Hence, this protein is very important for energy production in *Mycoplasma* species, and the SNP in the pyruvate kinase-encoding gene may lead to the attenuation of *M. bovis*.

The different molecular functions of transketolase (Mbov_0212) include metal ion binding and transferase activity, and it is a key enzyme in the non-oxidative branch of the pentose phosphate pathway that transfers a two-carbon glycolaldehyde unit from a ketose donor to aldose-acceptor sugars (Jores et al., 2009). Moreover, transketolase is an immunodominant membrane protein that may be used as a biomarker for the serological diagnosis of contagious agalactia caused by *M. mycoides* subsp. *capri* (Corona et al., 2013). Hence, this protein may be involved in the immunogenicity and virulence of *M. bovis*.

ABC transporter proteins have different functions, including ATP binding, ATPase activity, and catalyzing the transmembrane movement of substances, such as importing sugars, amino acids, peptides, metal ions, and phosphates, and effluxing toxins, drugs, and proteins (Higgins et al., 1986). The Mbov_0742 gene, which contained a SNP only in strain P180, encodes a glycerol ABC transporter protein. The Mbov_0581 gene, which had an indel only in strain P180, encodes a multiple sugar ABC transporter protein. Both genes are considered to encode virulence-related factors in the VFDB. In addition, the Mbov_0134 and Mbov_0018 genes encoding ABC proteins responsible for the uptake of simple sugar and spermidine/putrescine were found to have SNPs in all three attenuated strains.

NADH dehydrogenase (Mbov_0299) is considered to be a virulence-related factor. It generates energy by transferring electrons from NADH (oxidation) to quinine. NADH and NADPH are possibly essential for the growth of *M. suis* (Guimaraes et al., 2011). In *Mycobacterium tuberculosis*, a mutant lacking NuoG, a subunit of the type I NADH dehydrogenase complex, exhibited attenuated growth *in vivo* (Blomgran et al., 2012). Hence, this gene mutation might be significantly related to the further attenuation of *M. bovis* strain P180.

Phosphate acetyltransferase (PTA) (Mbov_0567) catalyzes the formation of acetyl phosphate and acetyl CoA, which are used in many metabolic pathways, e.g., in taurine and hypotaurine metabolism, pyruvate metabolism, propanoate metabolism, and methane metabolism (Figure S5). Acetyl-phosphate regulates various cellular processes, including cell division, outer membrane protein expression, osmoregulation, and biofilm development. Moreover, acetyl phosphate is required for the activation of the Rrp2-RpoN-RpoS pathway, which serves as a global signal in bacterial pathogenesis by activating virulent genes (Xu et al., 2010). Moreover, *Salmonella enterica* serovar Typhimurium (Kim et al., 2006) and *Vibrio cholerae* (Chiang and Mekalanos, 1998) PTA mutants were shown to exhibit impaired growth and attenuated virulence. In addition, because of a SNP in Mbov_0567, a proline residue was converted to threonine, resulting in polarity change that might be significant in virulence attenuation.

In addition, membrane proteins influence cell shape, cell division, motility, and adhesion to host cells, and they are thought to be integrally involved in the pathogenesis of mycoplasmas. As is known, adhesion and invasion are generally considered to be virulence-associated processes. *M. bovis* can adhere to and invade epithelial cells and immune cells. Membrane lipoproteins, such as Vsps, enolase, and Vpmax, play significant roles in the adhesion of *M. bovis* to host cells (Burki et al., 2015). Five genes encoding membrane proteins were shown to have SNPs, and four of them were predicted to encode secretory proteins with signal peptides, including Mbov_0393, Mbov_0525, Mbov_0579, and Mbov_0797. Among them, Mbov_0797 encodes a Vsp, and it was predicted to be a virulence-related factor, Mbov_0579 was predicted to encode the ADP-ribosyltransferase CDTa, which contains functional domains of the community-acquired respiratory distress syndrome toxin of *M. pneumoniae* (Kannan et al., 2014).

### Alternation of H_2_O_2_ production in the attenuated strains

H_2_O_2_ is thought to be a significant virulence related factor in *M. pneumoniae, M. mycoides*, and *Mycoplasma ovipneumoniae*. Secondary metabolites are considered to play significant roles in the pathogenesis of some *Mycoplasma* species (Pilo et al., 2005; Hames et al., 2009). For example, H_2_O_2_ was demonstrated to be a major virulence-related factor that leads to cell death and lipid peroxidation in *M. pneumoniae* (Hames et al., 2009) and *MmmSC* (Pilo et al., 2005). Moreover, activation of glycerol utilization and overproduction of H_2_O_2_ occurred during intracellular infection with *Mycoplasma gallisepticum* (Matyushkina et al., 2016). Although an *in vitro* H_2_O_2_ assay for *M. bovis* field strains showed that variations in H_2_O_2_ production did not correlate with *M. bovis* virulence (Schott et al., 2014), *in vitro* passaging an *M. bovis* strain resulted in decreased levels of H_2_O_2_ production (Khan et al., 2005).

**Figure 4:**
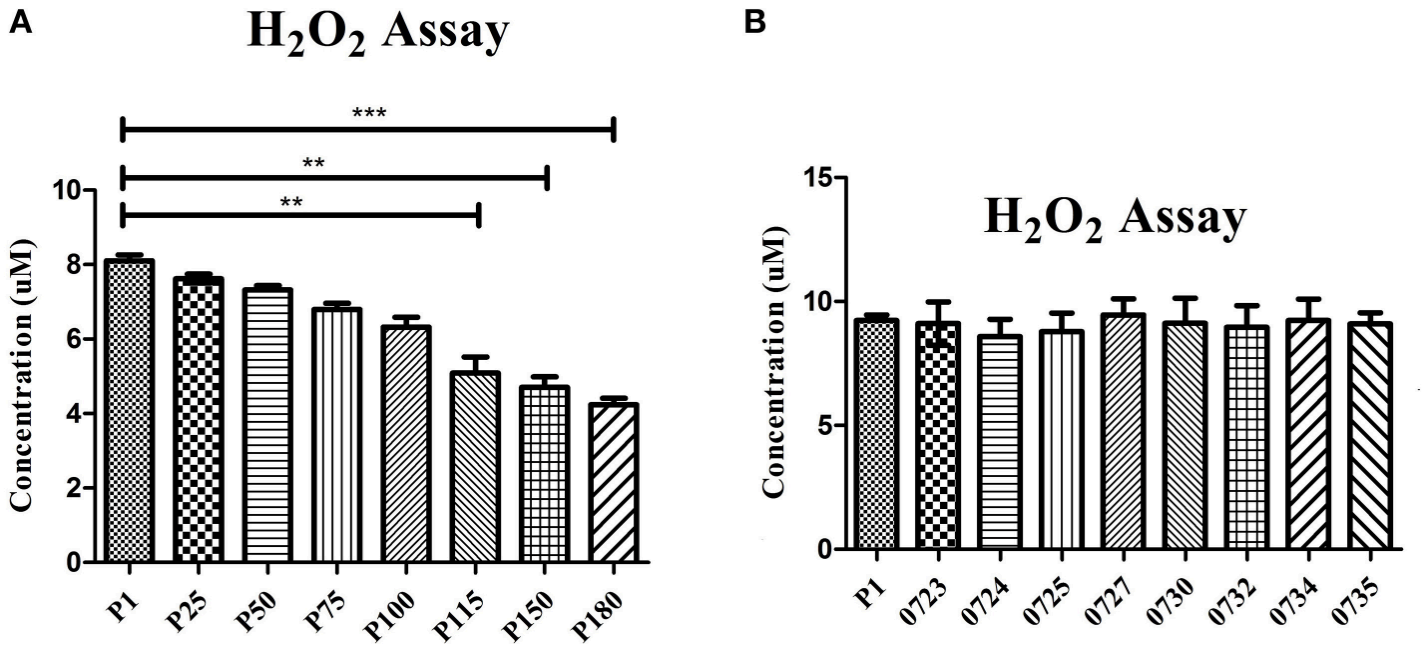
**Production of H_**2**_O_**2**_ by 10^**8**^ cells/ml determined after a 30-min incubation with 10 mM glycerol**. Significant differences between groups are highlighted with asterisks. **(A)** Comparison of the wild-type *M. bovis* HB0801 strain and strains that were subjected to different numbers of passages during *in vitro* growth. **(B)** Comparison of the wild-type *M. bovis* HB0801 with different transposon-disrupted mutants during *in vitro* growth. These transposon-disrupted mutants were produced using the virulent *M. bovis* HB0801 and a Tn4001 transposon. Gene numbers of *M. bovis* given are as follows. 0723 = Mbov_0723, 0724 = Mbov_0724, 0725 = Mbov_0725, 0727 = Mbov_0727, 0730 = Mbov_0730, 0732 = Mbov_0732, 0734 = Mbov_0734, and 0735 = Mbov_0735.

**Figure 5:**
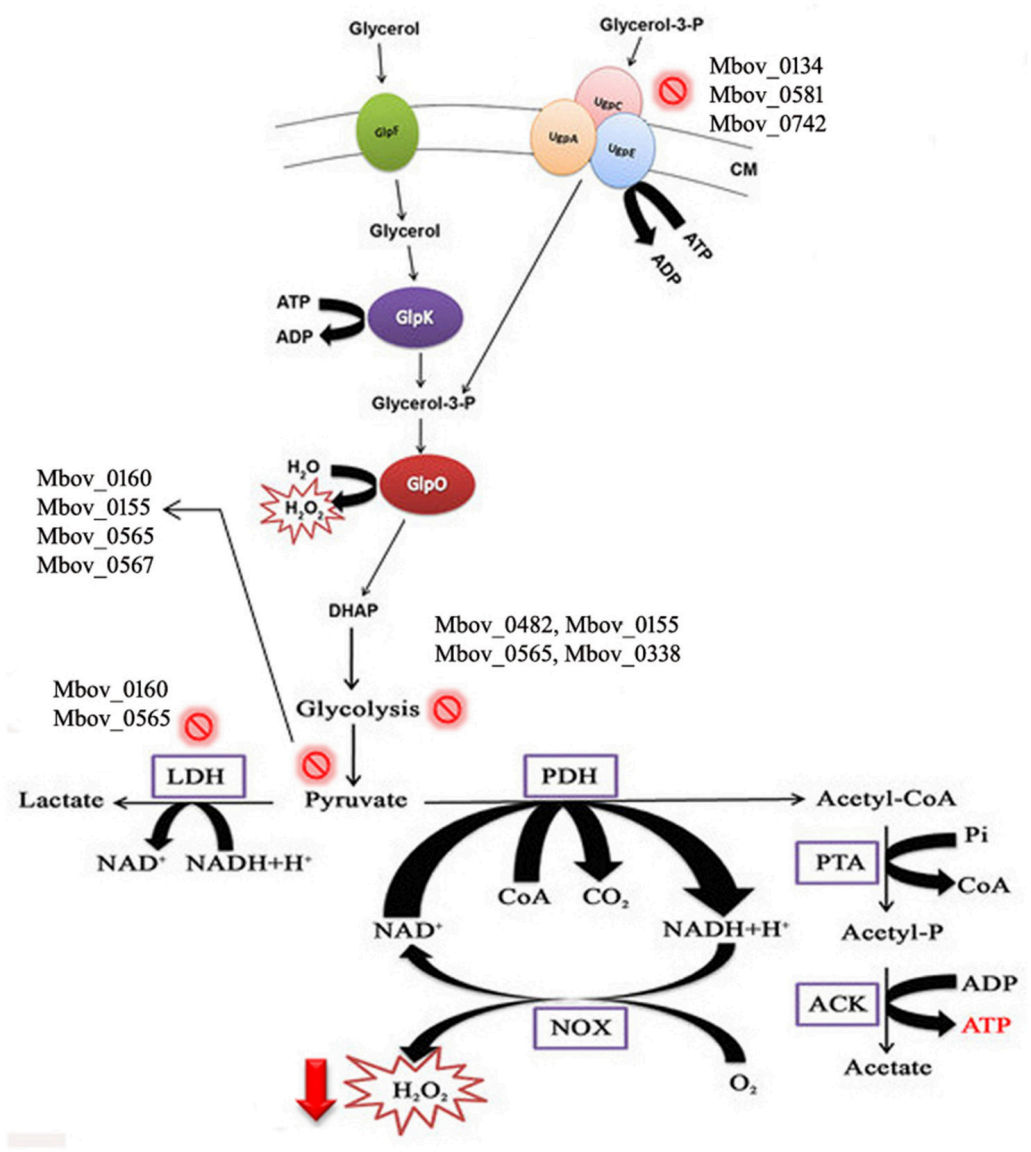
**Schematic illustration of H_**2**_O_**2**_ production in mycoplasmas affected by multiple pathways, including glycerol metabolism**. Glycerol is transported into the cell by the GlpF membrane transporter. Glycerol is phosphorylated to glycerol-3-P by GlpK (glycerol kinase). Moreover, glycerol-3-phosphate is also recycled through ABC transporters (UgpA, C, and E). Glycerol-3-phosphate is converted to dihydroxyacetone phosphate (DHAP) by oxidoreductase GlpO. H_2_O_2_ is secreted during and after the whole process. Mutations in the genes of mycoplasmas related to these metabolic pathways lead to decreased H_2_O_2_ production. CM, cell membrane. The 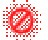 sign shows the mutations in the pathways, and mutated genes are listed with this sign.

To confirm that H_2_O_2_ production was affected by the mutations in the attenuated strains, H_2_O_2_ production was tested in the wild-type strain HB0801 (P1), strains of various passages (25, 50, 75, 100, 115, 150, and 180), and eight mutants specific to the genes in the 14.2-kb deleted region (Mbov_0723, Mbov_0724, Mbov_0725, Mbov_0727, Mbov_0730, Mbov_0732, Mbov_0734, and Mbov_0735). The results showed a decreasing tendency of H_2_O_2_ production following increasing numbers of passages in *M. bovis*. The differences in H_2_O_2_ production between passage 1 and the serially passaged strains began to be statistically significant from passage 115 and onward (*p* < 0.01) (Figure 4A). Interestingly, other researchers measured the production of H_2_O_2_ by *in vitro* passaged strains including the 50th, 100th, and 200th passages of *M. bovis*, and they showed a similar decreasing tendency of H_2_O_2_ production with increasing numbers of passages (Khan et al., 2005). Random transposon mutagenesis was used recently to generate *M. bovis* mutants (Sharma et al., 2014). With the availability and further improvement of these techniques, it should be possible to obtain detailed information about the interactions of *M. bovis* with its host in the near future. To differentiate the effects resulting from the 14.2-kb deleted region and the SNPs in related genes on H_2_O_2_ production, transposon-disrupted mutants were further characterized. However, there was no significant difference in H_2_O_2_ production by the mutants compared with the P1 strain (Figure 4B). Therefore, these genes in the 14.2-kb deleted region might not affect H_2_O_2_ production, or a single gene could only contribute slightly to H_2_O_2_ production. In fact, other genes outside the 14.2-kb deleted region that have SNPs and indels might be associated with altered H_2_O_2_ production via the impairment of various pathways (Figure 5), such as ABC transporters (Figure S6), carbon metabolism (Figure S7), biosynthesis of amino acids (Figure S8), glycolysis/gluconeogenesis (Figure S9), and pyruvate metabolism (Figure S10).

## Conclusions

We first sequenced the genomes of three attenuated *M. bovis* strains, HB0801-P115, HB0801-P150, and HB0801-P180, with various levels of virulence, and we performed a comprehensive comparative genomics analysis of these strains. The results not only enrich the basic genomic data for further research of *M. bovis*, but they also provide guidance for exploring the molecular mechanisms of *M. bovis* virulence and pathogenesis.

## Author contributions

MR performed experiments, analyses, and wrote the manuscript. JQ, XZ, and HC performed experiments. HM, FK, GZ, and MZ performed analyses. AG, HCC, YC, and CH designed experiments and revised the manuscript.

## Funding

This work was supported by the National Natural Science Foundation of China (grant Nos. 31661143015 and 31302111), the Special Fund for the Chinese Agricultural Research System (Beef/yaks) (grant No. CARS-38), the National Key Research and Development Program of China (grant No.2016YFD0500906) and the Special Fund for National Distinguished Scholars in Agricultural Research and the Technical Innovative Team.

### Conflict of interest statement

The authors declare that the research was conducted in the absence of any commercial or financial relationships that could be construed as a potential conflict of interest.
