# Supplementary material for: Comparative Genomics of Mycoplasma bovis Strains Reveals That Decreased Virulence with Increasing Passages Might Correlate with Potential Virulence-Related Factors
Source: Front Cell Infect Microbiol. 2017 May 11;7:177. doi: 10.3389/fcimb.2017.00177 (PMC5426083; doi:10.3389/fcimb.2017.00177)

Table S1 Primers for confirmation of SNPs and indels in P115, P150 and P180 strains compared to HB0801 strain of *Mycoplasma bovis*

**Location sites of the SNPs and indels in HB0801**	**Change**	**Sequences of primers（5'-3'）**	**Direction of primers**
17328	C-A	TAACCGACCAAGAAATCCAAGG	Sense
		TAATCCATGCTTGTGTCTGTCG	Reverse
58153	G-A	AAGCTGGTATTCAGGATGTTAAAGAT	Sense
		TAATGAAGAATGAGTCCGAGAAGTT	Reverse
120789	C-T	ACACCATTAGTGGCTGCTAGAT	Sense
		ATACTGAAGGACGGGCATTAAC	Reverse
151187	C-A	AGTTGTCAAAGAATTCGATGGC	Sense
		TTGTGAGGCGGCAAGTCT	Reverse
158099	poly A	TGTATGTGAACACTGTGGCTTT	Sense
		ACCTTGTGATTGTGCAACACT	Reverse
183285	C-T	TAGCTGTTGGTGAGTATGATGG	Sense
		TGGTATGGACTGTGTTGTTGTT	Reverse
187262	C-T	TAGGTATGTTTCAGCCAGTAGC	Sense
		AATGACTGCAACACCGAAGG	Reverse
241786	C-T	ACCTTGAATTGCCGAGTTATGT	Sense
		ATATAGTTAGCCCACACGTATGC	Reverse
247443	C-T	ACTAAGGTAAGGTTCGCTGATTC	Sense
		GGATCGTTTGATGCTTCTATTGC	Reverse
284709	G-T	CAACAGGTATGAGAATAGGTGAGT	Sense
		GCCATTGTAGCTAAAGTTCTTCTG	Reverse
313924	poly A	TTGCCTAAGATGCTTGTACCTT	Sense
		TGCCTTCTCGTCAACATTTCC	Reverse
350175	G-A	AATCAGCAGCATGTTCACCTT	Sense
		TGCACAACACCTAGTTGAGTTAG	Reverse
388007	C-A	CTACCTAAGCAGTCACCATCAG	Sense
		AGTAGCCTTAAAGCAGCAACAT	Reverse

399903	A-*	GTGTTAAGGAAGTTGATGTTCC	Sense
		TCCATCTTTAGTGTAAGCCGAC	Reverse
402407	poly CTAGT	AATCTGCTTGCTTGACTGTGG	Sense
		CGCCTCTAGTTCTGGTGTTATTG	Reverse
414907	Del TC	ACAATCTATAATGCAAACCGCTTAT	Sense
		GAGATAATTGGCGATAGTGTTGTC	Reverse
418864	C-A	TGCTGATAGTTGAAAGGCTGAT	Sense
		ATCTTGGCGTATAATAAGCAGC	Reverse
423698	poly C	AAGCATCAATTCATTGTTGGAACT	Sense
		ATTCATTATGGCAGTGTATTATCCG	Reverse
426282	poly T	TCTAATAAGTATTTAGGGTCGTCATT	Sense
		CCATTAATAGCTGCATCATGTCAA	Reverse
457329	C-A	GAGAAAGCATTAACTGGTGTTG	Sense
		TGCATTGTATAACGTTTGGC	Reverse
472982	C-T	AAGCTAGAGCACAAGGTGACTT	Sense
		CAACAACATCGCCAATTCCATTAG	Reverse
553603	poly T	GGCTTTAGGAATAAGTGCTGAT	Sense
		CTAATATAGGGCATGAGTGAAACC	Reverse
604926	poly T	GGCGTATTAGGTGGATATTGAGAG	Sense
		CGAATATAGCAGTTACGGCATCT	Reverse
607195	poly T	GATTTAATCCTGGGCCAAAGC	Sense
		TTAGAGACATACTAGCAGCCTTAC	Reverse
609729	C-T	TTGGTAGTTGTTGGTGATGAGT	Sense
		CCAGAGTAATAACAGGTCCTACAC	Reverse
613519	poly T	ACTTCAGGGCTAGGATTAGTTTCT	Sense
		AGTTATGTTCTTGCTGGTTCTGAG	Reverse
618377	C-T	GATGGCTTCGCTTCAAATGTG	Sense
		AGTCTCTGCTAATAGTGTTGTCC	Reverse
620722	poly G	GCCACCGTTTGTATCTTCTAATAG	Sense
		ATTGCTGCGGCTTGTAATTCT	Reverse
628787	C-T	GTTGGTTGTTGGTGACTTAGTTC	Sense

		ACATCAGGATTAGTTCAGCTTCC	Reverse
638676	G-A	TTCTGTACGGAATAGTTCTTGGAC	Sense
		CCTGTATCGCCATTAGGAAGC	Reverse
656673	poly A	ACTTGCCATTGTGATTTGAACC	Sense
		AGATCATTGTGGCTAGGAACATC	Reverse
667404	C-T	AGAATGTCTGTGTGAACCTTAGC	Sense
		TTAGAGCATCAGTATTAGGCGAAC	Reverse
669593	C-A	GCCACTGAAAGAGGCTTAAATG	Sense
		CTCCTTGTAATATTGCACCAACTG	Reverse
684890	A-T	TGATTAATGCAGATGCTAATGACTT	Sense
		TCGCCACTAACATCAGTACCT	Reverse
691444	C-A	TTAAGATGGCTGGTGCTGTG	Sense
		AGCAAGCAATATGCTAGGACTT	Reverse
742189	C-A	GCTTCCTGTGCTAGTTACTTCTC	Sense
		CGATGTTATAATGGCTGCAATAGC	Reverse
743651	G-A	AACGATCAACTAAGTCGCCATT	Sense
		GTAGTGACCACAGCACATTTATTG	Reverse
768061	poly G	CATCGTGTGTCTGTTAATTCTTCTAT	Sense
		GCTTCTGTTGTGTCTGCTCTT	Reverse
777220		GCTAAGCTCCACAACTCTATATGC	Sense
		AGGTAGTGGCTTGACCAACTC	Reverse
798981	Del TC	TTGTGTTGCAGCCGCATT	Sense
		GGTAATGAGATAATTGGCGATAGC	Reverse
833489	G-A	AAGCAAACTGGCAAGATTATCC	Sense
		AACTGAGGCAATGAATTGTTCG	Reverse
845529	C-A	GGGCACTATCTATTTGAATGAG	Sense
		AAAGGCAGAACCATCTGAAT	Reverse
848007	C-A	GGTATTGTTAAGCCTGATGTTCC	Sense
		GCTGTTAGATGAATTGGTTGCT	Reverse
871124	T-C	ACCCACTTTCTTCTATCAGTTCCT	Sense
		AACGCATCAAGCAGCAACG	Reverse

895729	T-C	CGCTTCTTTGCCTCTTGTTG	Sense
		GCAACTAGAAGACAATCCTGAAC	Reverse
901268	G-C	TGGATGATGGTGGAAGAACTCA	Sense
		GCCAGTGATTGCTTACGGTAA	Reverse
925890	G-T	CAACACTAACAATTCCACTCCTAC	Sense
		TTGAAGCCAATGAAGCTACTGA	Reverse
926716	T-G	AGAAGACGAAGAAGACGAAGATTC	Sense
		TGGTTTGTCCGCTTCTAGTTTC	Reverse
957308	T-C	TCTAAATCCTAGTTCGTCAGCAAAT	Sense
		GGTAAAGAAGAGCAAAGGGTTGA	Reverse
959470	poly T	CCAAATGCTGCTAATCCAAAGTTAT	Sense
		GAGGCAACAATGAATGATGAAGTG	Reverse
984777	indel G	TCACTAATTGTTCACTCGCT	Sense
		GGGAACAGATTCTAGCAATCC	Reverse

Table S2 The comparison between the reference proteins and their paralogues

Gene number	Query coverage (%)	Positives (%)	Gaps (%)	Identities (%)
**For DNA methyltransferase where Mbov_0727 was taken as reference**				
Mbov_0202	61	52	14	34
Mbov_0413	62	43	20	27
Mbov_0526	71	41	16	26
**For Putative lipoproteins where Mbov_0518 was taken as reference**				
Mbov_0024	9	66	0	58
Mbov_0049	8	53	20	32
Mbov_0084	9	60	17	36
Mbov_0111	24	47	5	25
Mbov_0116	21	53	0	46
Mbov_0119	11	50	2	35
Mbov_0145	17	48	8	29
Mbov_0156	21	45	13	20
Mbov_0177	29	57	0	29
Mbov_0179	8	56	0	58
Mbov_0186	23	55	4	35
Mbov_0188	15	50	8	28
Mbov_0193	12	56	2	27
Mbov_0260	19	57	7	38
Mbov_0274	11	61	0	44
Mbov_0275	14	41	26	30
Mbov_0283	9	55	8	38
Mbov_0289	16	62	0	27
Mbov_0290	22	51	6	41
Mbov_0291	25	73	11	54
Mbov_0296	9	67	0	50
Mbov_0347	12	52	0	41
Mbov_0350	16	58	17	38
Mbov_0374	24	50	0	32
Mbov_0393	14	55	13	38
Mbov_0436	6	35	0	23
Mbov_0449	10	57	7	46
Mbov_0461	1	66	0	44
Mbov_0468	30	48	18	31
Mbov_0473	9	61	0	35
Mbov_0475	9	56	0	44
Mbov_0477	27	47	1	32
Mbov_0489	35	42	12	29
Mbov_0505	15	60	6	40
Mbov_0515	98	62	4	45
Mbov_0525	6	56	0	44
Mbov_0536	15	66	0	50
Mbov_0537	6	57	0	43
Mbov_0538	8	69	0	38
Mbov_0546	18	59	21	38
Mbov_0548	29	53	17	36
Mbov_0570	24	46	10	24
Mbov_0578	7	81	0	55
Mbov_0585	21	66	0	28
Mbov_0592	3	52	0	32
Mbov_0654	1	88	0	44
Mbov_0659	10	51	4	33
Mbov_0664	6	45	16	32
Mbov_0672	3	50	0	31
Mbov_0674	17	69	3	38
Mbov_0682	6	58	2	39
Mbov_0689	3	58	0	29
Mbov_0696	13	53	0	32

Mbov_0699	19	59	9	41
Mbov_0729	15	47	0	25
Mbov_0732	12	50	7	36
Mbov_0739	5	57	0	34
Mbov_0803	23	50	0	24
Mbov_0838	24	57	7	29
Mbov_0856	5	62	6	31
**For ATP binding proteins where Mbov_0581 was taken as reference**				
Mbov_0018	28	54	3	34
Mbov_0033	40	54	4	29
Mbov_0034	55	44	12	32
Mbov_0114	30	42	3	22
Mbov_0115	30	52	0	29
Mbov_0134	49	64	5	42
Mbov_0135	1	87	0	63
Mbov_0136	10	53	0	36
Mbov_0271	20	61	12	34
Mbov_0552	22	47	16	30
Mbov_0594	26	54	1	31
Mbov_0595	33	56	0	38
Mbov_0742	33	69	0	44
Mbov_0843	34	60	0	32
Mbov_0853	24	66	0	42
**For Thioredoxin where Mbov_0832 was taken as reference**				
Mbov_0100	28	52	0	26
Mbov_0576	28	53	0	27
**For Alcohol Dehydrogenase where Mbov_0338 was taken as reference**				
Mbov_0312	79	43	11	26
Mbov_0353	98	76	0	54

Note: FASTA sequences of proteins were aligned with our reference protein by NCBI BLAST in order to check the percentage of identity between paralogues. The reference protein is the protein which is found in our deleted region or SNP data.

Table S3 The KEGG pathways enriched by *M.bovis* genes with deleted region and SNPs.

**Term**	**ID**	**Input number**	**Background number**	**P-Value**	**Corrected P–Value**	**Genes involved in pathways**
Microbial metabolism in diverse environments	mbi01120	11	37	0.0082	0.1399	Mbov_0155, Mbov_0160, Mbov_0206, Mbov_0212, Mbov_0328, Mbov_0338, Mbov_0482, Mbov_0565, Mbov_0567, Mbov_0722, Mbov_0723,
Metabolic pathways	mbi01100	11	101	0.6771	0.6771	Mbov_0155, Mbov_0206, Mbov_0212, Mbov_0328, Mbov_0338, Mbov_0482, Mbov_0565, Mbov_0567, Mbov_0722, Mbov_0723, Mbov_0727,
Biosynthesis of antibiotics	mbi01130	6	23	0.0772	0.3504	Mbov_0155, Mbov_0206, Mbov_0212, Mbov_0338, Mbov_0482, Mbov_0565
Biosynthesis of secondary metabolites	mbi01110	6	34	0.2455	0.3508	Mbov_0155, Mbov_0206, Mbov_0212, Mbov_0338, Mbov_0482, Mbov_0565
Carbon metabolism	mbi01200	5	22	0.1499	0.3504	Mbov_0155, Mbov_0206, Mbov_0212, Mbov_0482, Mbov_0567
Pyruvate metabolism	mbi00620	4	11	0.0653	0.3504	Mbov_0155, Mbov_0160, Mbov_0565, Mbov_0567
Biosynthesis of amino acids	mbi01230	4	14	0.1147	0.3504	Mbov_0155, Mbov_0206, Mbov_0212, Mbov_0482
Glycolysis / Gluconeogenesis	mbi00010	4	17	0.1764	0.3504	Mbov_0155, Mbov_0338, Mbov_0482, Mbov_0565
Pentose phosphate pathway	mbi00030	3	10	0.1524	0.3504	Mbov_0206, Mbov_0212, Mbov_0522
Purine metabolism	mbi00230	3	20	0.452	0.5488	Mbov_0155, Mbov_0206, Mbov_0522
ABC transporters	mbi02010	3	24	0.5645	0.5998	Mbov_0134, Mbov_0581, Mbov_0742
Phosphotransferase system (PTS)	mbi02060	2	5	0.1656	0.3504	Mbov_0722, Mbov_0723
Ascorbate and aldarate metabolism	mbi00053	2	6	0.2061	0.3504	Mbov_0722, Mbov_0723
Cysteine and methionine metabolism	mbi00270	2	6	0.2061	0.3504	Mbov_0565, Mbov_0727
Propanoate metabolism	mbi00640	2	7	0.2476	0.3508	Mbov_0565, Mbov_0567
Methane metabolism	mbi00680	2	8	0.2894	0.3785	Mbov_0482, Mbov_0567
RNA degradation	mbi03018	1	5	0.4929	0.5586	Mbov_0482

Note: ID is the code for respective pathway. Input numbers are the genes working together in particular pathway while background numbers are the total numbers of genes present in the particular pathway.

Figure S1: The PCR rsults for the confirmation of deleted region confirmed that the fragment was deleted in passage 115 and onward. The putative lipoprotein (Mbov_0732) present in the deleted region was selected for PCR with the forward primer (AGCGACCAAAATACTAGAC) and reverse primer (TCGTTGCCACTGTATTCA). 1-12：Negative, P69, P68, P67, P66, P65, P64, P63, P62, P61, P60, P59; 13-32: Negative, P71, P72, P73, P74, P76, P77, P78, P79, P80, P82, P83, P84, P86, P87, P88, P89, P90, P91, P92; 33-40: P94, P97, P100, P103, P106, P109, P112, P115; M: DNA Marker DL1000

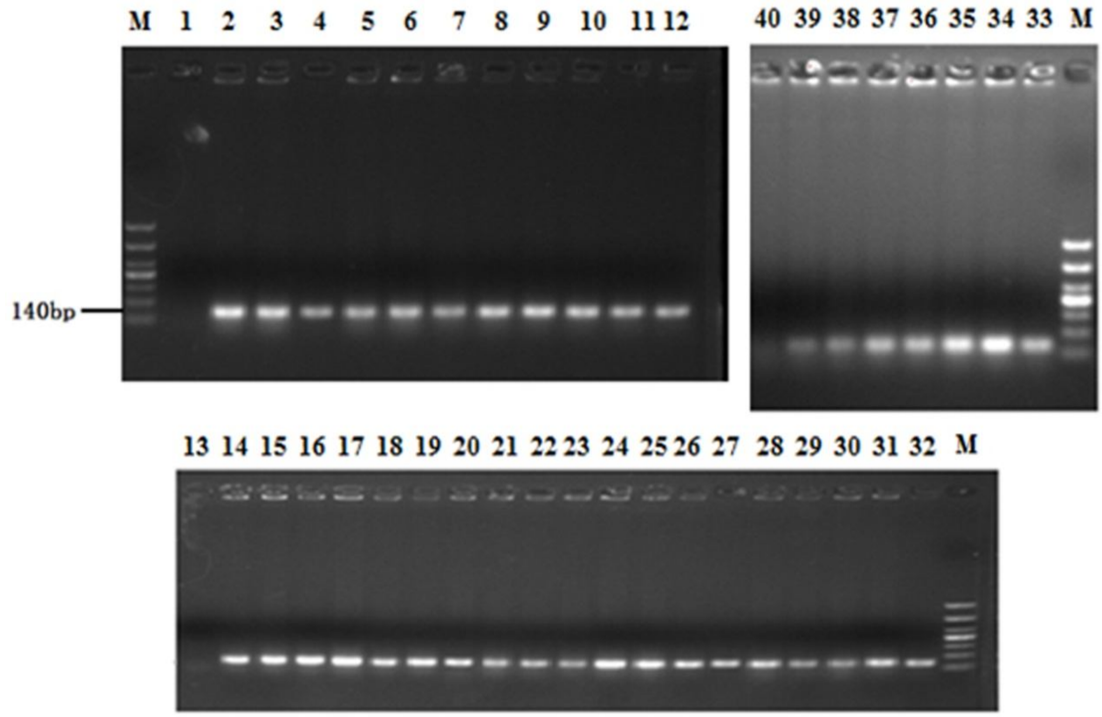

Figure S2: Involvement of Mbov_0722 and Mbov_0723 proteins in ascorbate and aldarate metabolism. The enzymes convert L-ascorbate to L-ascorbate-6P.

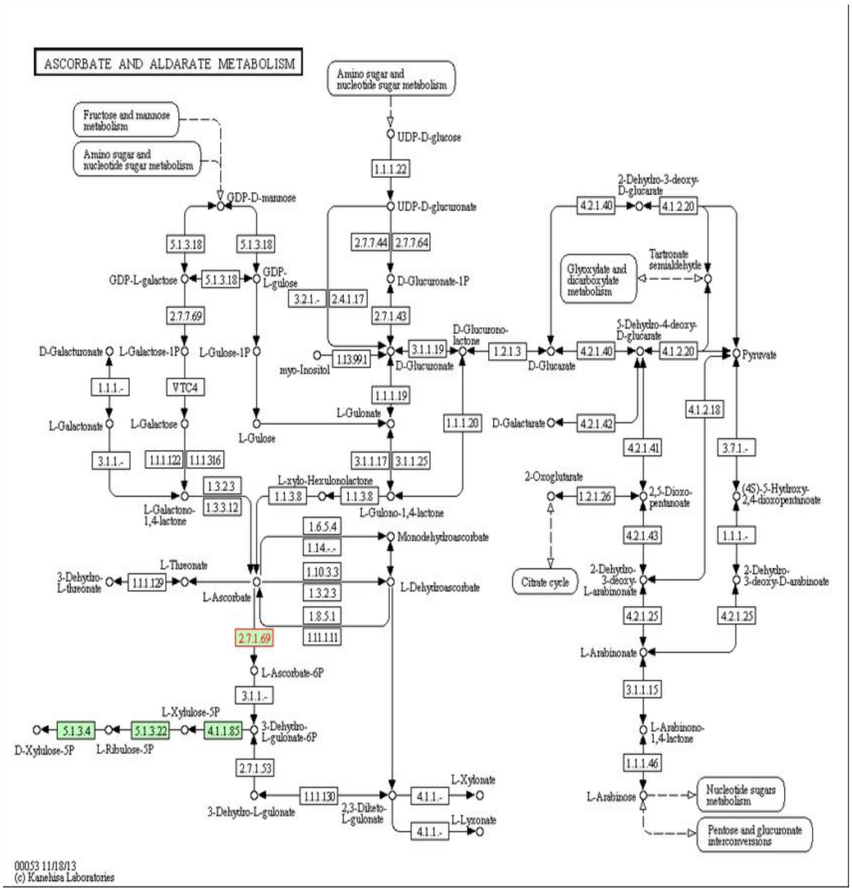

Figure S3: Involvement of Mbov_0482 protein in glycolysis/gluconeogenesis metabolism where it catalyzes the reversible conversion of 2-phosphoglycerate into phosphoenolpyruvate

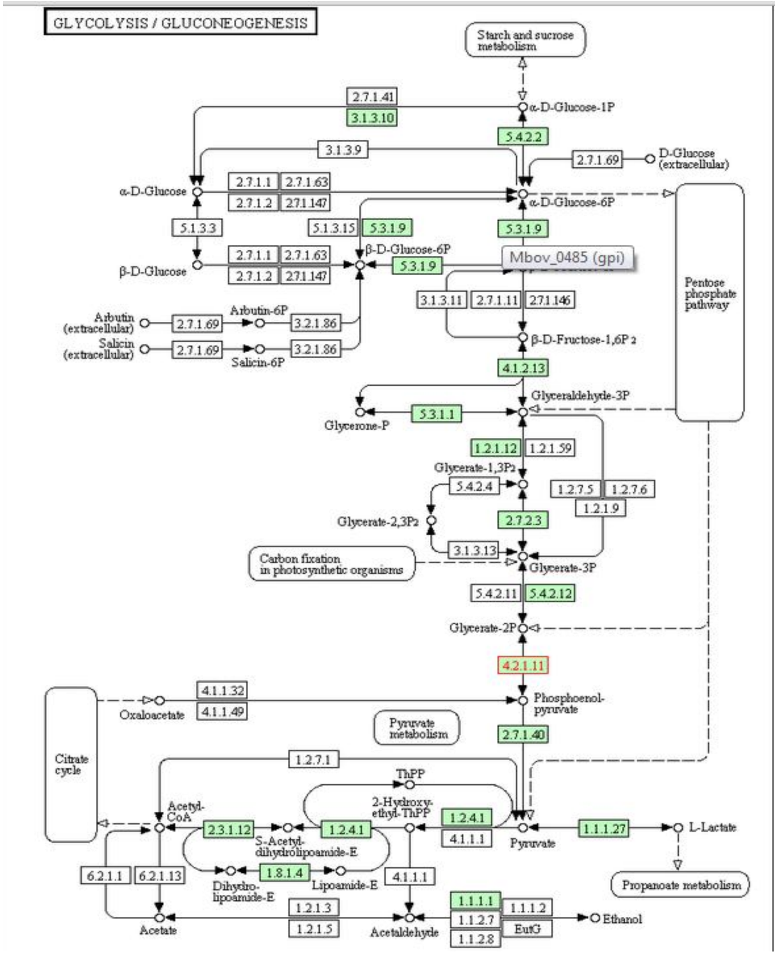

Figure S4: Mbov_0155 protein catalyzes the process of conversion of phosphoenolpyruvate to pyruvate

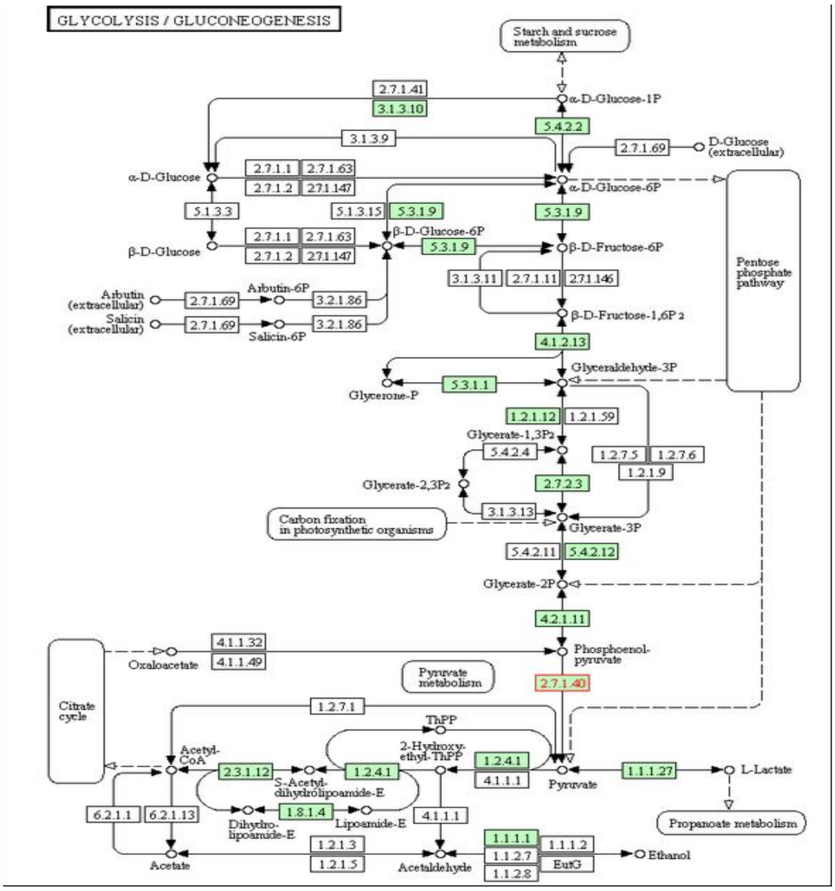

Figure S5: Mbov_0567 protein catalyzes the formation of Acetyl phosphate and Acetyl Co A

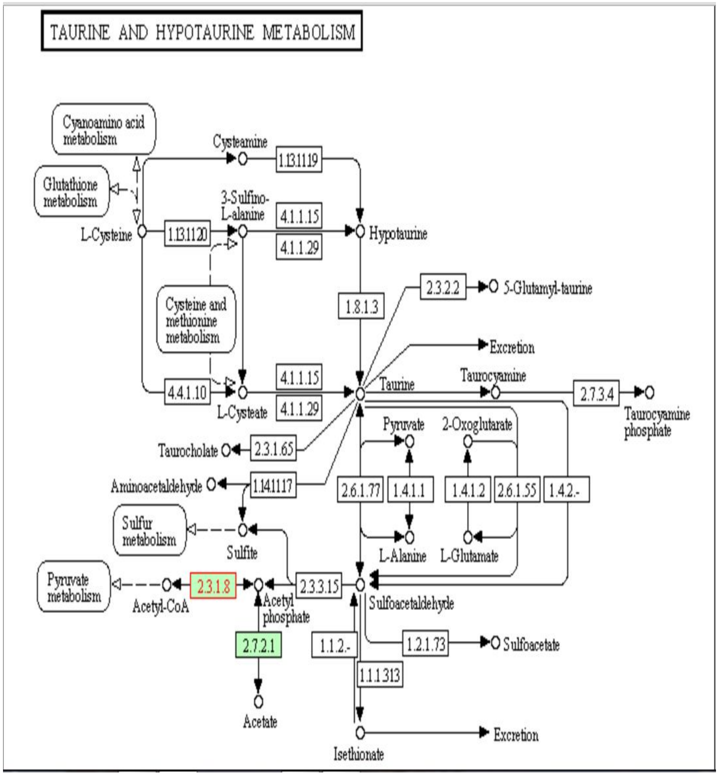

Figure S6: Multiple genes working in same ABC transporters metabolic pathway. KEGG pathway id is mbi02010. PotA = Mbov_0134. MalK = Mbov_0581, Mbov_0742. MsmX, MsmK, SmoK, AglK, MsiK = Mbov_0581, Mbov_0742.

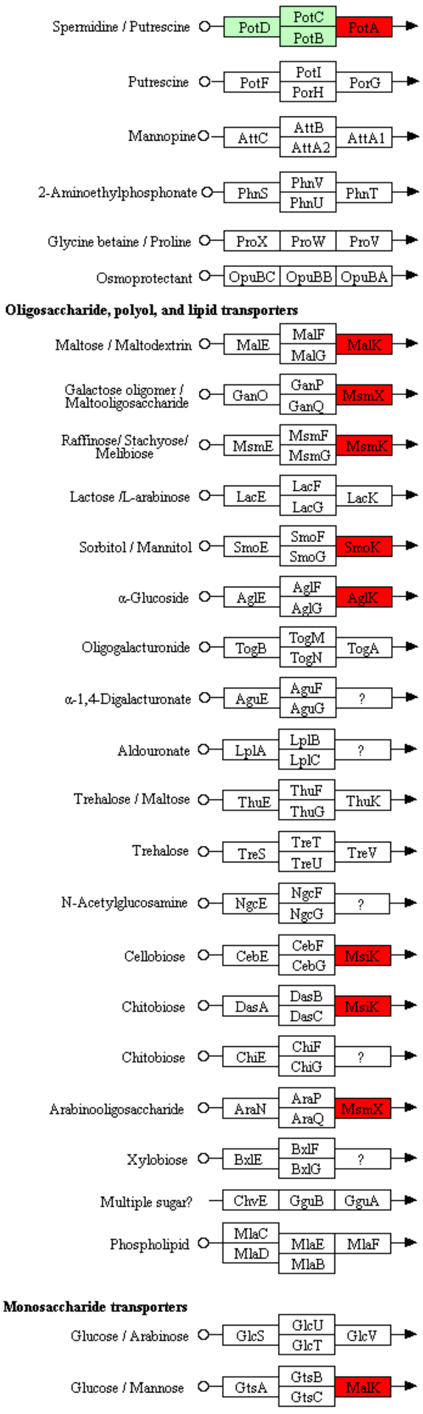

Figure S7: Multiple genes working in same carbon metabolism metabolic pathway. KEGG pathway id is mbi01200. Ribose-5P to PRPP = Mbov_0206. Glycerate-2P to phosphoenolpyruvate = Mbov_0482. Phosphoenolpyruvate to pyruvate = Mbov_0155. Others red arrows = Mbov_0212. Acetyl phosphate to acetyl co-A = Mbov_0567.

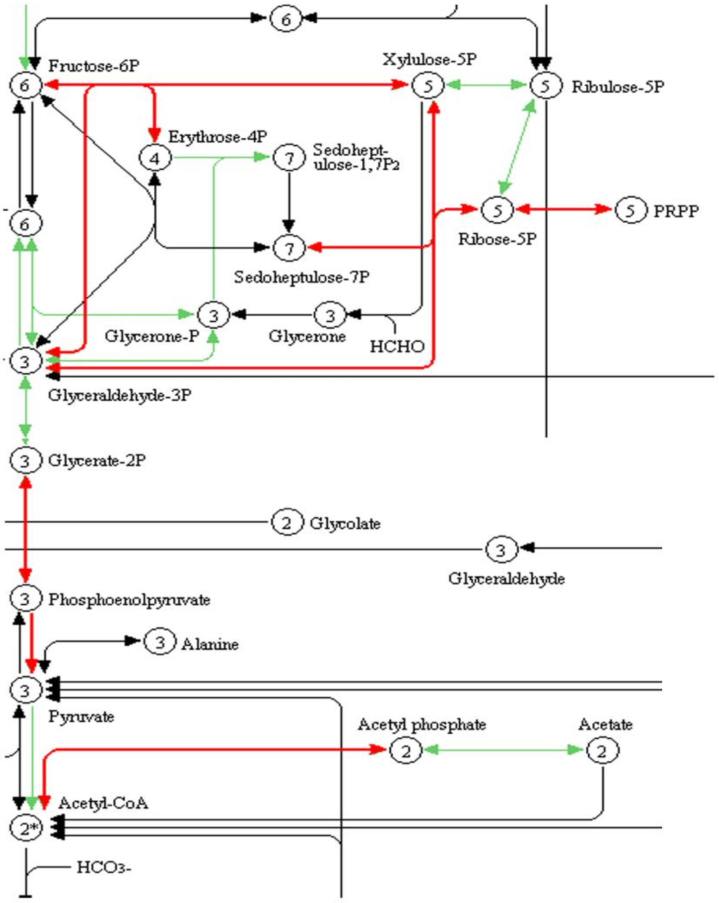

Figure S8: Multiple genes working in same biosynthesis of amino acids metabolic pathway. KEGG pathway id is mbi01230. Ribose-5P to PRPP = Mbov_0206. Glycerate-3P to phosphoenolpyruvate = Mbov_0482. Phosphoenolpyruvate to pyruvate = Mbov_0155. Others red arrows = Mbov_0212

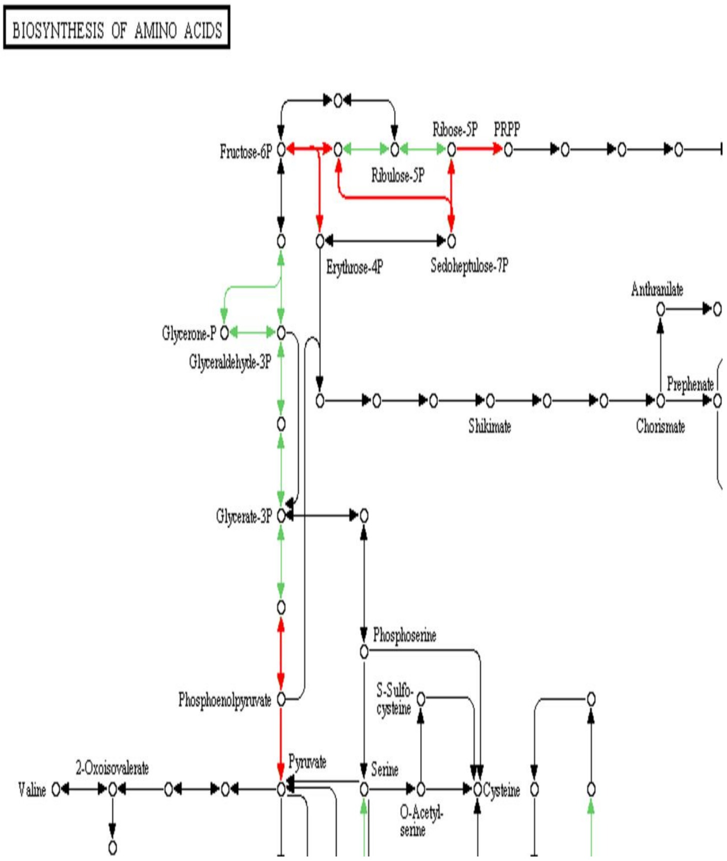

Figure S9: Multiple genes working in same Glycolysis/Gluconeogenesis metabolic pathway. KEGG pathway id is mbi00010. 4.2.1.11 = Mbov_0482. 2.7.1.40 = Mbov_0155. 1.1.1.27 = Mbov_0565. 1.1.1.1 = Mbov_0338.

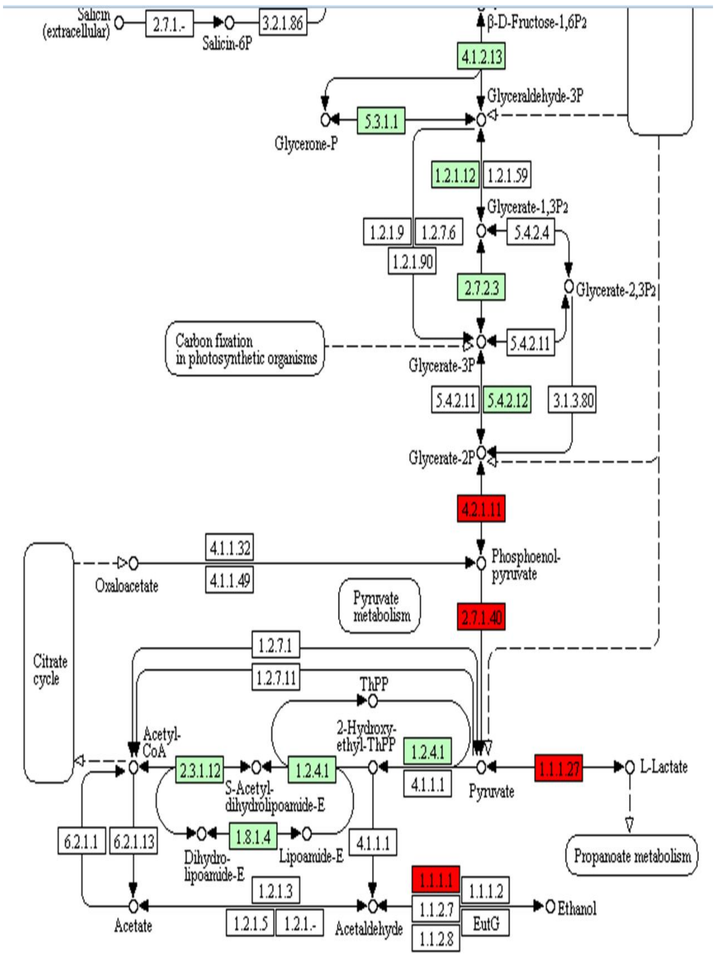

Figure S10: Multiple genes working in same pyruvate metabolism metabolic pathway. KEGG pathway id is mbi00620. 1.1.1.28 = Mbov_0160. 2.7.1.40 = Mbov_0155. 1.1.1.27 = Mbov_0565. 2.3.1.8 = Mbov_0567.

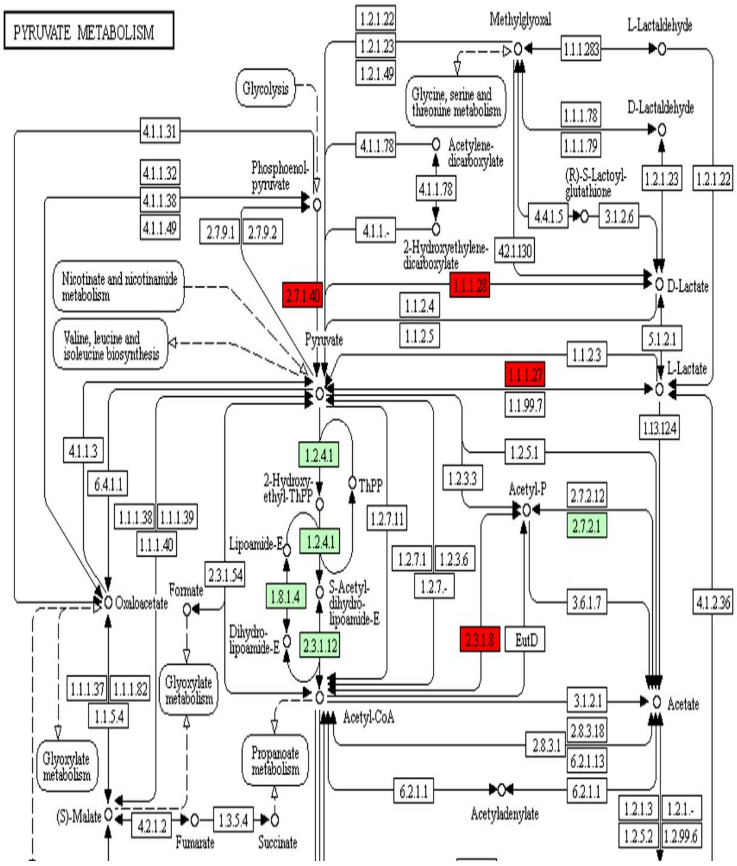

Supplement: Supplementary file 1 [file DataSheet1.PDF]
